# Asparagine deprivation enhances T cell antitumour response in patients via ROS-mediated metabolic and signal adaptations

**DOI:** 10.1038/s42255-025-01245-6

**Published:** 2025-03-05

**Authors:** Hsuan-Chia Chang, Chung-Ying Tsai, Cheng-Lung Hsu, Tzong-Shyuan Tai, Mei-Ling Cheng, Yu-Ming Chuang, Hsiang-Yu Tang, Kun-Ju Lin, Jia-Jin Chen, Szu-Han Chang, Yi-Ching Ko, Yu-Wen Chi, Hsuan Liu, Bertrand Chin-Ming Tan, Chia-Rui Shen, Chih-Wei Yang, Ping-Chih Ho, Huang-Yu Yang

**Affiliations:** 1 Graduate Institute of Biomedical Sciences, College of Medicine, Chang Gung University, Taoyuan, Taiwan; 2 Kidney Research Center and Department of Nephrology, Chang Gung Memorial Hospital, College of Medicine, Chang Gung University, Taoyuan, Taiwan; 3 Division of Hematology-Oncology, Department of Internal Medicine, Chang Gung Memorial Hospital, Chang Gung University, Taoyuan, Taiwan; 4 School of Medicine, Chang Gung University, Taoyuan, Taiwan; 5 Advanced Immunology Laboratory, Chang Gung Memorial Hospital, Taoyuan, Taiwan; 6 Department of Biomedical Sciences, College of Medicine, Chang Gung University, Taoyuan, Taiwan; 7 Clinical Metabolomics Core Laboratory, Chang Gung Memorial Hospital, Taoyuan, Taiwan; 8 Metabolomics Core Laboratory, Healthy Aging Research Center, Chang Gung University, Taoyuan, Taiwan; 9 Department of Oncology, University of Lausanne, Lausanne, Switzerland; 10 Ludwig Institute for Cancer Research, University of Lausanne, Epalinges, Switzerland; 11 Healthy Aging Research Center, Department of Medical Imaging and Radiological Sciences, College of Medicine, Chang Gung University, Taoyuan, Taiwan; 12 Department of Nuclear Medicine and Molecular Imaging Center, Chang Gung Memorial Hospital, Taoyuan, Taiwan; 13 Molecular Medicine Research Center, Chang Gung University, Taoyuan, Taiwan; 14 Department of Cell and Molecular Biology, College of Medicine, Chang Gung University, Taoyuan, Taiwan; 15 Division of Colon and Rectal Surgery, Lin-Kou Medical Center, Chang Gung Memorial Hospital, Taoyuan, Taiwan; 16 Research Center for Emerging Viral Infections, Chang Gung University, Taoyuan, Taiwan; 17 Department of Neurosurgery, Lin-Kou Medical Center, Chang Gung Memorial Hospital, Taoyuan, Taiwan; 18 PhD Program in Biotechnology Industry, College of Medicine, Chang Gung University, Taoyuan, Taiwan; 19 Center of Molecular and Clinical Immunology, Chang Gung University, Taoyuan, Taiwan; 20 Department of Ophthalmology, Linkou Chang Gung Memorial Hospital, Taoyuan, Taiwan; 21 Department of Health Policy and Management, Johns Hopkins Bloomberg School of Public Health, Baltimore, MD USA

**Keywords:** Immunology, Cancer therapy, Metabolism

## Abstract

Preclinical studies have shown that asparagine deprivation enhances T cell antitumour responses. Here we apply compassionate use of l-asparaginase, usually employed to treat blood malignancies, on patients with recurrent metastatic nasopharyngeal carcinoma. The use of l-asparaginase notably enhances immune-checkpoint blockade therapy in patients by strengthening CD8^+^T cell fitness. Our study shows that this combination is a promising avenue for clinical application and provides further mechanistic insight into how asparagine restriction rewires T cell metabolism.

## Abstract (web summary)

The compassionate use of l-asparaginase in patients suffering from nasopharyngeal carcinoma substantially strengthens the efficacy of immune-checkpoint blockade therapy by enhancing T cell fitness.

## Main

Immune-checkpoint inhibitors (ICIs) have emerged as a promising therapeutic strategy to re-evoke antitumour immunity by targeting immune-checkpoint pathways^1–3^; however, the efficacy of ICIs can be limited by the suppressive nature of the tumour microenvironment that prevents T cell tumour infiltration and promotes T cell dysfunction^4,5^. Emerging evidence underscores the potential synergistic effects and improved clinical outcomes by combining ICIs with traditional chemotherapy drugs in the treatment of various cancers^6,7^. Previous studies reported that asparagine deprivation detrimentally affects protein synthesis and the phosphorylation of Src-family tyrosine kinase Lck, thereby suppressing T cell activation^8^. On the other hand, prolonged asparagine restriction has recently been reported to induce stress response signals, including ATF4 and NRF2 expression, which impact metabolic reprogramming and lift up T cell antitumour activity^9^. However, the discrepancy on T cell activation and function in response to asparagine deprivation in these reports remains unresolved due to the lack of mechanistic understandings. Moreover, it remains unclear whether asparagine deprivation induced by asparaginase treatment can be exploited for re-evoking T cell-mediated antitumour immunity in humans.

**Fig. 1:**
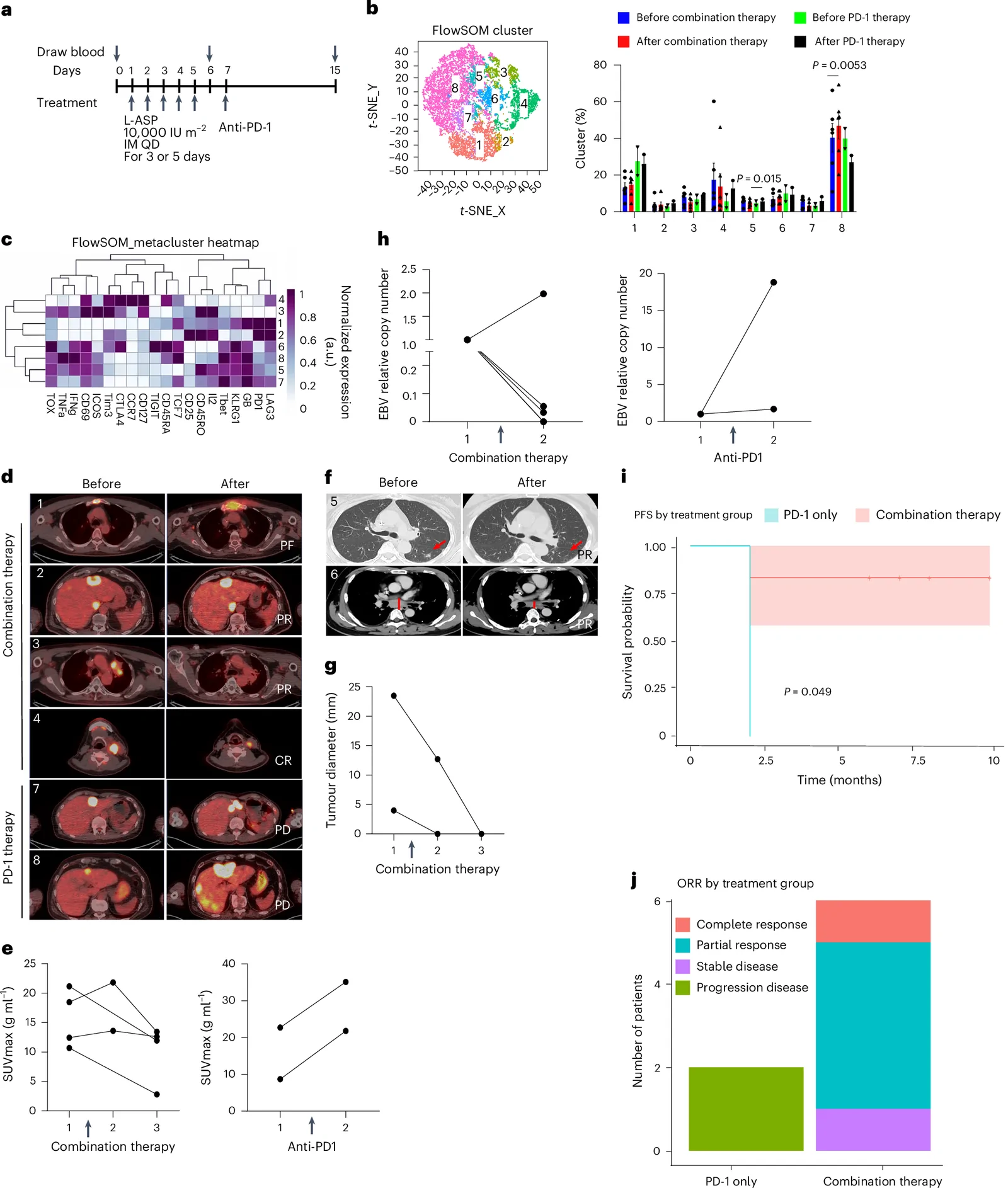
Asparaginase boost PD-1 blockade-induced antitumour immunity. **a**, Schematic diagram illustrating the design of a clinical trial for patients with NPC. IM QD, intramuscularly once a day; l-ASP, l-asparaginase. **b**, *t*-distributed stochastic neighbour embedding (*t*-SNE) analysis of CD3^+^CD8^+^T cells from peripheral blood mononuclear cell (PBMC) populations (*n* = 8) identifies distinct clusters of T cells. Percentage of CD3^+^CD8^+^T cells from different days in each FlowSOM cluster. **c**, FlowSOM clusters, with a heat map of effector molecule mean fluorescence intensity (MFI) overlaid onto the *t*-SNE analysis. **d**, PET images of representative patients from the combination therapy group (*n* = 4) and anti-PD-1 monotherapy group (*n* = 2) before and after treatment. **e**, Changes in maximum SUV (SUVmax) in tumours following treatment. **f**, CT scans of two representative patients before and after combination therapy. Red arrows indicate tumour locations post-treatment. **g**, Tumour diameter measurements for **f** over the course of combination therapy (*n* = 2). **h**, Plasma EBV relative DNA copy number distribution in patients with metastatic NPC conduct combination therapy (*n* = 6) and PD-1 therapy (*n* = 2). **i**, PFS by treatment group. **j**, ORR by treatment group. CR, complete response; PD, progressive disease; PR, partial response; PF, progression-free. All data are mean ± s.e.m. and were analysed by one-tailed, paired Student’s *t*-test (**b**). NS, not significant. Source data

**Extended Data Fig. 1:**
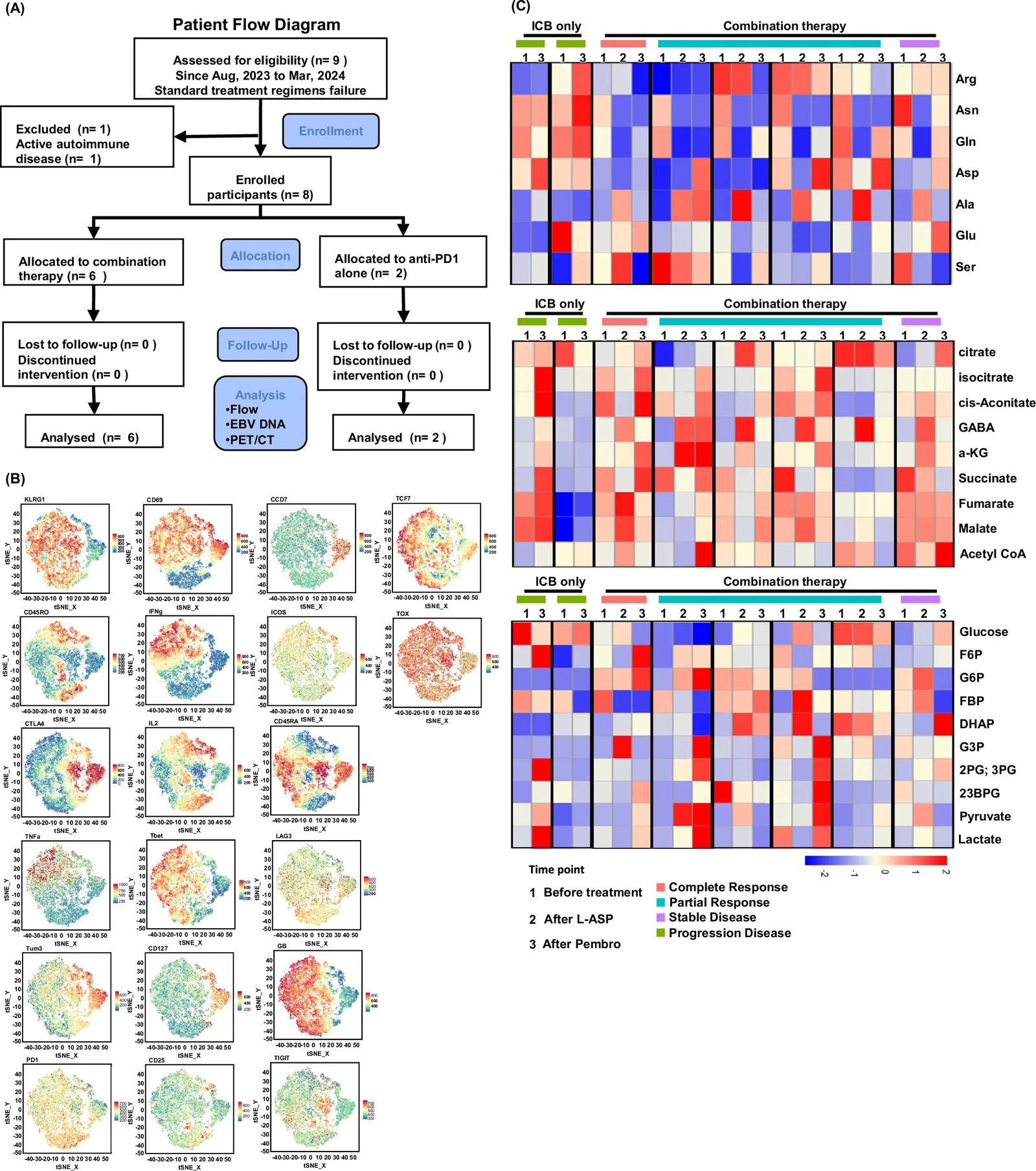
Immunoprofiling of CD8 + T cells and metabolomics dynamics after therapy. (**A**)Flow diagram illustrates the process of patient enrolment, allocation, follow-up, and analysis in the compassionate-use study. A total of nine patients were assessed for eligibility from August 2023 to March 2024. One patient was excluded due to active autoimmune disease, resulting in eight enroled participants. Allocation was based on the patients’ preferences, with six patients opting for combination therapy involving L-asparaginase and anti-PD-1, and two patients opting for anti-PD-1 monotherapy. All participants were followed up, with no losses to follow-up or discontinued interventions. The analysis included flow cytometry, EBV DNA quantification, and PET/CT imaging. (**B**) t-SNE plots generated using FlowSOM to illustrate the expression levels of various immune markers in Fig. 1b. (**C**)The metabolomic profiles of patients before and after treatment. Each row represents a metabolite, and each column represents a patient sample (n = 8). The colours indicate the z-score normalized abundance of each metabolite. Source data

As programmed cell death protein 1 (PD-1) therapy only provides modest benefits for patients with recurrent metastatic (RM) nasopharyngeal carcinoma (NPC)^10,11^, we wondered whether asparaginase can be used for improving antitumour response in patients with NPC. We provided the combination treatment of asparaginase plus anti-PD-1 monoclonal antibody (mAb) as a compassionate therapy (Extended Data Fig. 1a) for six patients with RM-NPC, who received PD-1 therapy but still displayed progressive disease (Table 1). These patients received sequential asparaginase (daily injections for 3 or 5 days) followed by pembrolizumab, an anti-PD-1 mAb (single injection), and compared the results of patients receiving only anti-PD-1 mAb (Fig. 1a). We performed FlowSOM (Fig. 1b and Extended Data Fig. 1b) to better define the changes induced by treatments. We found that combination therapy led to a notable increase in cluster 8 (Fig. 1c), reflecting populations with heightened cytokine production and elevated activation markers. Concurrently, cluster 6-expressing lower cytokines and some inhibitory receptors also increased, suggesting an augmented population of effector and exhausted T cells after combination therapy. In the PD-1 group, cluster 5-expressing cytokines and activation were slightly elevated. In addition to profiling CD8^+^ T cells, we performed positron emission tomography (PET)-computed tomography (CT) imaging and found that combination therapy declined the standardized uptake value (SUV) signal (Fig. 1d,e). Of note, we found that combined treatment induced the appearance of a pseudotumor in the sternum and robust increase of the signal in the tumour. We also examined the tumour burden by CT scan in two patients receiving combined therapy and found that combined therapy decreased the tumour volume (Fig. 1f,g). Most notably, the Epstein–Barr virus (EBV) DNA copy number, which is correlated with NPC burden^12^, showed a robust decline after the combination treatment but not after PD-1 treatment alone (Fig. 1h), indicating that the combination therapy effectively altered T cell activation and suppressed tumour progression. Kaplan–Meier analysis for progression-free survival (PFS) and the objective response rate (ORR) indicated robust improvement in the combination therapy group (Fig. 1i,j). The combination therapy group demonstrated higher complete and partial response rates compared with the PD-1-only group, where all patients experienced disease progression. A serum metabolomic analysis before and after treatment showed that l-asparaginase reduced asparagine and glutamine levels, with aspartic acid and glutamic acid levels increasing concurrently (Extended Data Fig. 1c). After anti-PD-1 treatment, glutamine levels partially recovered, whereas asparagine levels did not, indicating a temporary glutaminase effect of l-asparaginase. Our results suggest that asparaginase enhances the efficacy of anti-PD-1 therapy by promoting more robust immune cell activation, providing experimental evidence for the potential of combining asparaginase with anti-PD-1 to better harness host antitumour immunity. While these findings offer valuable insights into the use of l-asparaginase as a salvage therapy for RM-NPC, the limitations should be acknowledged, including the small sample size and the absence of sequential monitoring of tumour progression. As this study was conducted under compassionate-use conditions rather than as a formal clinical trial, stricter reporting and monitoring protocols were not possible, which should be taken into account when interpreting the results.

**Table 1:** Baseline characteristics and clinical outcomes of patients receiving combination therapy versus anti-PD-1 monotherapy

	Combination therapy						Anti-PD-1 alone	
**Case**	1	2	3	4	5	6	7	8
**Age, years**	50–54	55–60	35–39	50–54	30–34	50–54	55–60	65–69
**Sex**	Male	Male	Male	Male	Male	Female	Female	Male
**NPC stage (initial)**	T4N1M0 stage IVa	T4N2M0 stage IVa	T4N2M1 stage IVb	T4N1M0 stage IVa	T1N2M0 stage III	T3N3M stage IVb	T3N2M1 stage IVb	T4N3M1a stage IVb
**NPC treatment (before asparaginase or ICI therapy)**	CCRT Gem + Cis Taxo + Cis Pembo (clinical trial)	Gem + Carbo T + Carbo Chemo + Pembo 5-FU	Pembo Gem +Cis Taxo	CCRT Cis + 5-FU Taxo + Cis Xeloda Pembo Chemo + Pembo Gem + Carbo	CCRT Gem + Cis Pembo (clinical trial)	Gem + Cis Taxo + Cis Cetuximab Pembo Taxo Chemo + Pembo	CCRT Gem + Cis Carbo Pembo Chemo + PemboTaxo	Gem + Cis Chemo + Pembo Taxo + Carbo Gem + Cis
**NPC stage (before asparaginase or ICI therapy)**	T0N0M1 stage IVb	T3N0M1 stage IVb	T4N2M1 stage IVb	T0N0M1 stage IVb	T0N0M1 stage IVb	T3N3M1 stage IVb	T4N2M1 stage IVb	T4N3M1 stage IVb
**Metastasis (before asparaginase or ICI therapy)**	Liver and bone	Liver	Liver	Lung, bone, lymph node and liver	Lymph node lung	Lung and bone	Liver and lung	Liver and bone
**Smoking**	Yes	Yes	No	No	Yes	No	No	No
**Betel nut**	Yes	No	No	No	Yes	No	No	No
**Underlying disease**	HBV	DM (diet control)	Nil	DM, HTN, gout	Nil	HBV	Nil	BPH
**Follow-up period after NPC diagnosis**	202104 diagnosis 202110 recurrence 202201 liver meta 202205 bone meta	202009 diagnosis 202208 liver meta	202209 diagnosis 202203 recurrence 202212 liver meta	201602 diagnosis 202203 recurrence	2021 diagnosis 2019 recurrence 2019 ICI	201911 diagnosis 2019 ICI 202003 lung meta	202205 diagnosis 202207 liver and lung meta	202302 diagnosis Liver and bone meta
**Follow-up period after asparaginase or ICIs therapy combination or monotherapy**^**a**^	202308 combination therapy	202312 combination therapy	202403 combination therapy	202403 combination therapy	202402 combination therapy	202403 combination therapy	202206-202301 Pembo 202303-202312 Chemo + Pembo	202302 Chemo + Pembo
**Image evaluation after combination therapy**	Decrease SUVmax	Decrease SUVmax	Decrease SUVmax	Decrease SUVmax	Decreased tumour length in CT	Decreased tumour length in CT	Increased SUVmax	Increased SUVmax
**Mortality**	202312 died							
**Image evaluation by iRECIST**	Immune-stable disease	Immune partial response	Immune complete response	Immune partial response	Immune partial response	Immune partial response	Unconfirmed progression	Unconfirmed progression

This table summarizes the demographic and clinical characteristics of patients with NPC treated with combination therapy (l-asparaginase and anti-PD-1) versus those treated with anti-PD-1 alone. BPH, benign prostate hypertrophy; DM, diabetes mellitus; HBV, hepatitis B virus; HTN, hypertension; CCRT, concurrent chemoradiotherapy; Cis, cisplatin; Gem, gemcitabine; Pembo, pembrolizumab; Taxo, taxotere; Carbo, carboplatin; iRECIST, immune Response Evaluation Criteria in Solid Tumours; 5-FU, 5-fluorouracil.

^a^Follow-up assessments started from the initiation of combination therapy or the first immune-checkpoint inhibitor therapy for patients receiving monotherapy alone. Follow-up continued until patient mortality.

**Extended Data Fig. 2:**
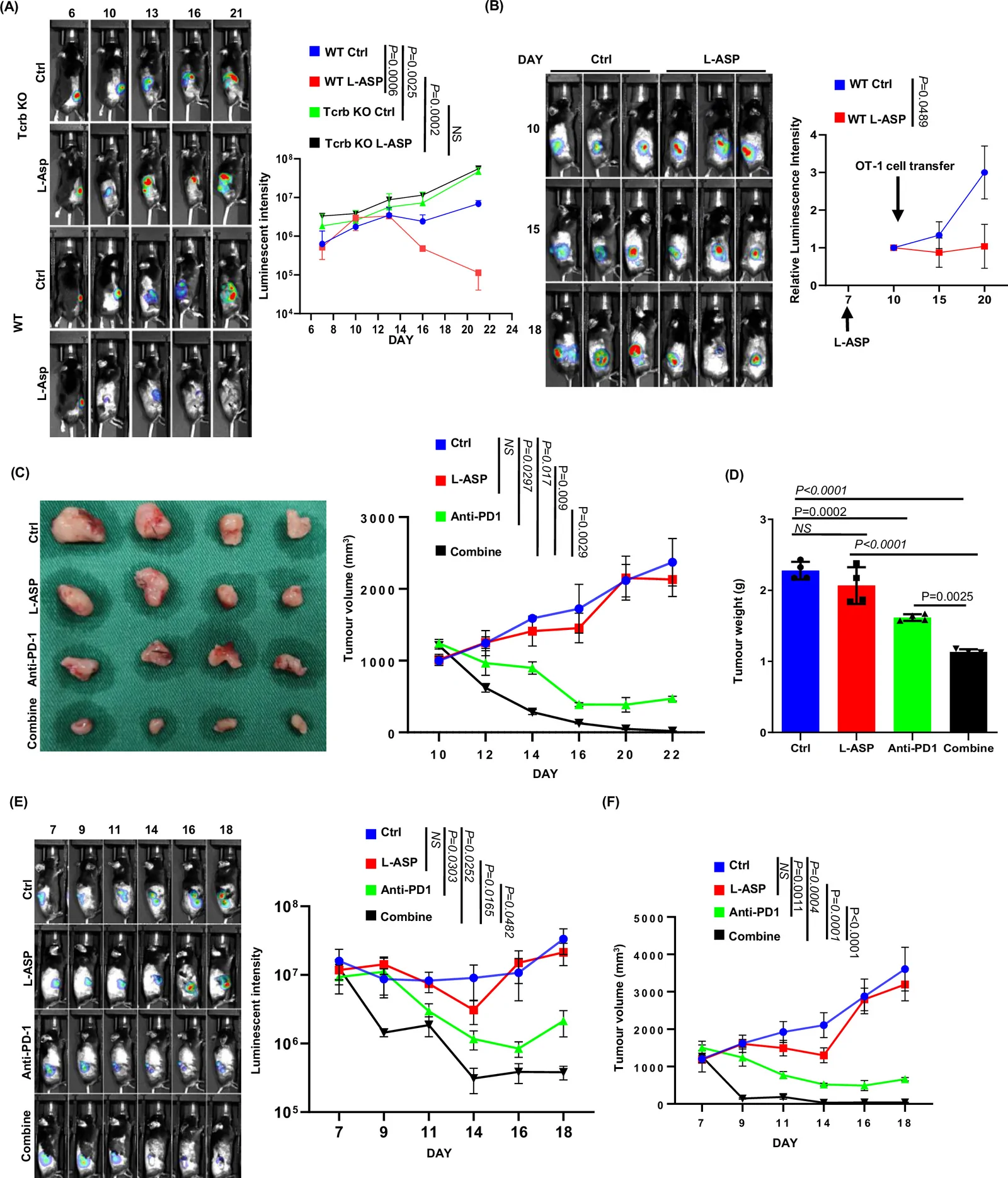
Asparaginase synergize with anti-PD-1 to elevate T cell antitumour responses. (**A**) B16-F10-Luc-bearing C57BL/6 and Tcrb KO mice were administered with vehicle or L-asparaginase (5 U/g) three days before tumour inoculation and then 3 times per week by intraperitoneal injection. The average luminescent intensity of photons emitted from each tumour in the images was quantified. Data are presented as mean ± SEM (n = 4 mice per group). (**B**) B16-OVA-bearing C57BL/6 mice were administered with vehicle or L-asparaginase (5 U/g) or PBS on day 7 after tumour inoculation and received 1.5 × 10^6 activated OTI T cells on day 10. The average luminescent intensity of photons emitted from each tumour in the images was measured. Error bars represent the standard error of the mean (SEM). Data represent one independent experiment (n = 3 mice per group). Error bars indicate SEM. (**C**) MTCQ1-bearing C57BL/6 were treated with either a vehicle, L-ASP, anti-PD-1, or a combination of L-ASP and anti-PD-1 starting on day 7 after tumour inoculation. The average tumour volume is emitted from each tumour group (left) and final tumour weight at the endpoint of the experiment (right)(n = 4 mice per group). (**D**) Tumour weight was measured at the end of the experiment. The average tumour volume, luminescent intensity, and tumour weight for each treatment group are shown (n = 4). (**E**) B16-OVA-bearing C57BL/6 mice were treated with either a vehicle, L-ASP, anti-PD-1, or a combination of L-ASP and anti-PD-1 starting on day 7 after tumour inoculation (n = 4 mice per group). (**F**) Final tumour weight at the endpoint of the experiment (n = 4 mice per group). All data are mean ± SEM and were analysed by one-tailed unpaired (**A**, **B**, **E**, **F**) Student’s t-test, Sidak multiple comparisons tests (**C**) and Tukey’s multiple comparisons test (**D**) with adjusted P value and data are cumulative results from at least three independent experiments. Source data

**Extended Data Fig. 3:**
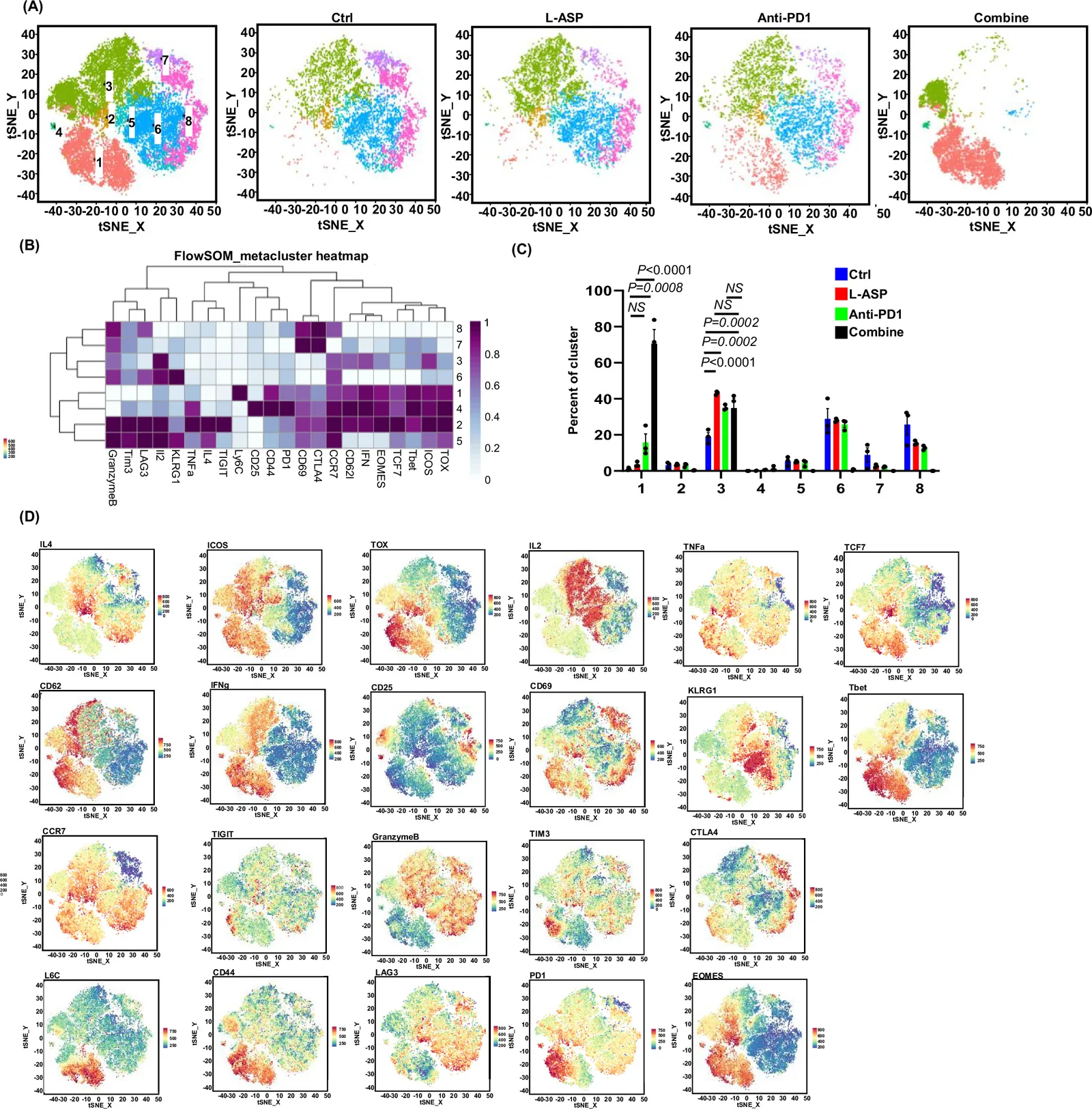
Asparaginase boost PD-1 blockade-induced CD8 + T cell activation. (**A**) t-SNE projections of CD45^+^ CD8^+^tumour-infiltrating lymphocytes (TILs), derived from tumours under different treatments (Ctrl, L-ASP, Anti-PD-1, and combination therapy). Clusters were identified by FlowSOM clustering, and the percentage of TILs in each cluster across different treatment groups is shown (n = 3). (**B**) FlowSOM clusters, with a heat map of effector molecule MFIs overlaid onto the t-SNE analysis. (**C**) Quantification of FlowSOM clusters across treatment groups, presented as the percentage of total Tcrb^+^ CD45^+^ CD8^+^ TILs per cluster. (**D**) t-SNE projections of selected protein expression from (**A**), showing the distribution of key markers related to T cell activation, exhaustion, and effector function. All data represent mean ± SEM from at least three independent biological replicates. Two-way ANOVA with Tukey’s multiple comparisons test was used in (**C**). Source data

**Extended Data Fig. 4:**
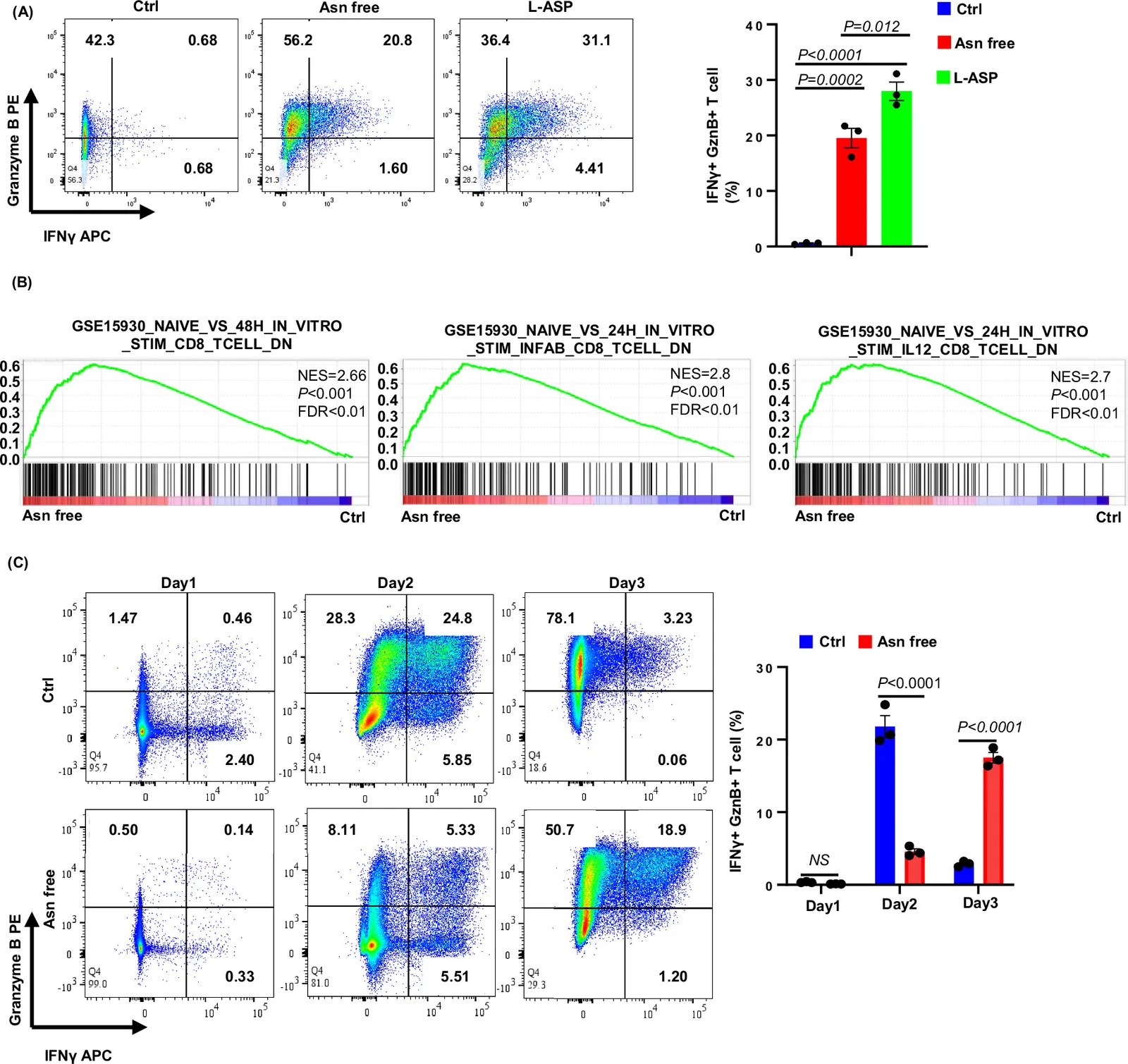
Asparagine deprivation enhances CD8 + T cell effector functions. Naïve CD8 + T cells were isolated from WT B6 mice and cultured under CD8 differentiation conditions using a medium without asparagine (Asn-free) or with 1U/ml asparaginase (L-ASP). (**A**) After three days of culture, the cells were re-stimulated with PMA and ionomycin for four hours in the presence of Golgistop. CD8 + T cells stained for IFN-γ and granzyme B (n = 3). (**B**) GSEA tracing for T cell activation in Asn-free and control cultured CD8 + T cell. ES, enrichment score; NES, normalized enrichment score; FDR, false discovery ratio. (**C**) human CD8 + T cells were isolated from human PBMCs using the Naive CD8 + T Cell Isolation Kit, human. CD8 + T cells cultured under control and asparagine-deprived conditions. After 3 days cultured, cells were then re-stimulated with PMA and ionomycin for 4 h with Golgistop. CD8 + T cells stained for IFN‐γ and granzyme B (n = 3). All data are mean ± SEM and were analysed by one-way ANOVA with Tukey’s multiple comparisons test (**A**) and two-way ANOVA with Sidak multiple comparisons tests (**C**) with adjusted P value and data are cumulative results from at least three independent experiments. Source data

Next, we aimed to delineate the underlying mechanisms and impact of combination therapy on CD8^+^ T cells by using mouse models. We observed that asparaginase treatment led to a robust reduction of tumour burden in wild-type mice but failed to suppress tumour cell growth in TCRb knockout (KO) mice (Extended Data Fig. 2a). Moreover, B16-OVA tumour-bearing mice treated with asparaginase before the transfer of tumour-specific OT-1 CD8^+^ T cells, completely impede tumour growth (Extended Data Fig. 2b), highlighting that the quality of tumour-reactive CD8^+^ T cells can be modulated in response to asparaginase treatment. We next examined the antitumour responses elicited by a single or combined treatment with asparaginase and anti-PD-1 in both B16-F10 melanoma and MTCQ1 oral carcinoma (OSCC)^13^ models. Indeed, the combination of asparaginase and anti-PD-1 induced the most potent antitumour responses compared with individual treatment in both tumour settings (Extended Data Fig. 2c–f). We further analysed CD8^+^ tumour-infiltrating lymphocytes in those treated mice with FlowSOM clustering (Extended Data Fig. 3a). Our result showed that combination therapy led to a strong increase in cluster 1, which exhibited increased expression of interferon (IFN)γ, CD44 and transcription factors (T-bet and EOMES), suggesting that combination therapy may potentiate CD8^+^ T cell activation in vivo (Extended Data Fig. 3b–d). Next, we found that both asparagine deprivation and asparaginase treatment effectively increased the ability of CD8^+^ T cells to express IFNγ and granzyme B (Extended Data Fig. 4a). Moreover, CD8^+^ T cells primed in asparagine-depleted culture displayed an enrichment in gene signatures related to T cell activation and responses induced by interleukin (IL)-12 and IFNβ (Extended Data Fig. 4b). Similarly, human CD8^+^ T cells activated in the asparagine-deprived medium for 3 days also displayed better ability to produce IFNγ and granzyme B (Extended Data Fig. 4c). Altogether, our results imply that asparaginase augments the efficacy of anti-PD-1 by fostering enhanced activation of immune cells and provide experimental evidence for applying asparaginase and anti-PD-1 combination therapy in better unleashing host antitumour immunity.

**Extended Data Fig. 5:**
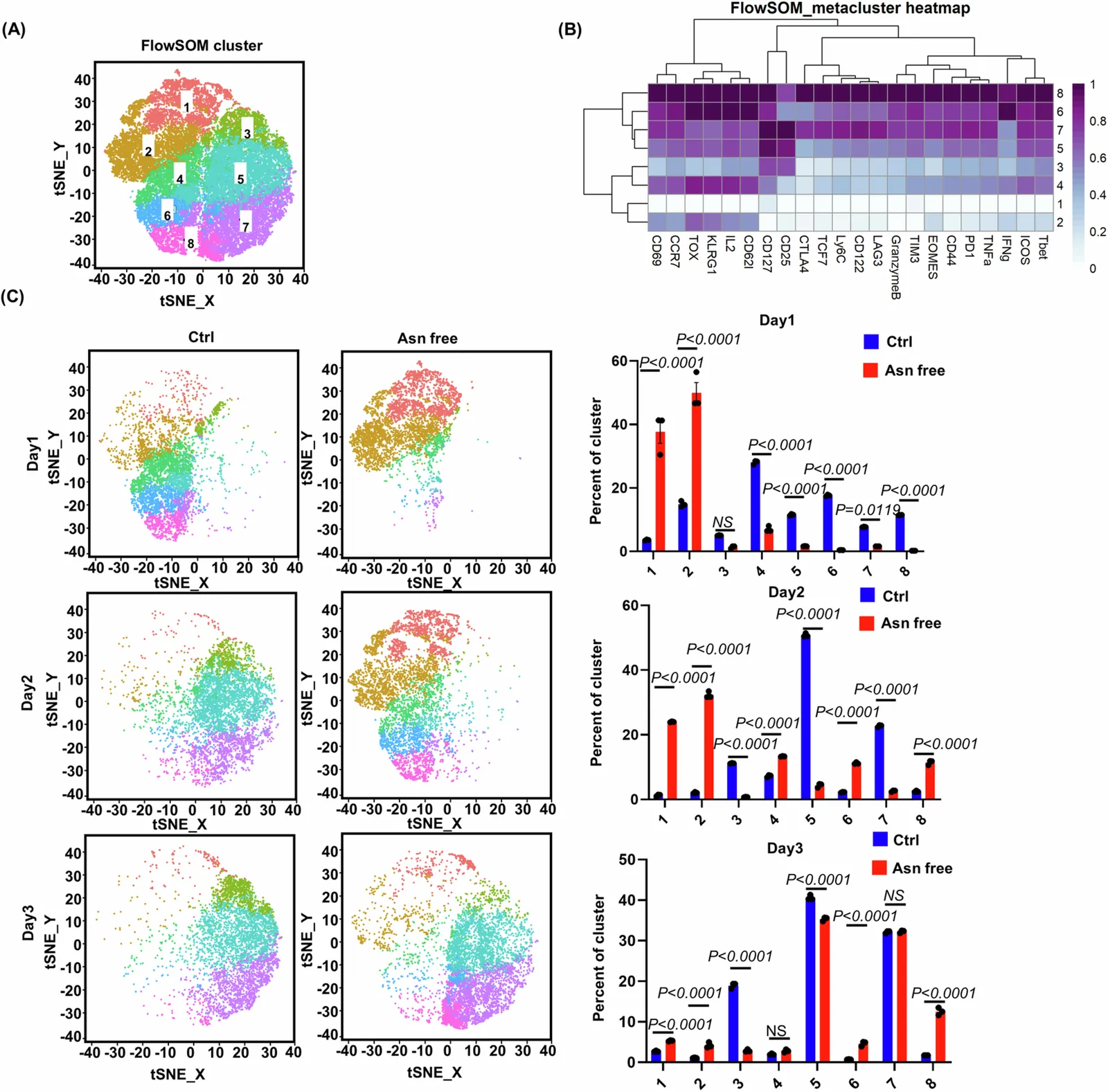
Prolonged asparagine deprivation enhances CD8 + T cell differentiation with more superior effector functions. Naïve CD8 + T cells were isolated from WT B6 mice and cultured under CD8 differentiation conditions using a medium without asparagine (Asn-free). For various durations, the cells were re-stimulated with PMA and ionomycin for four hours in the presence of GolgiStop, permeabilized, and stained for IFN-γ and granzyme B. (**A**) t-SNE analysis of CD8 + T cells in differentiation conditions using both control medium and asparagine-deprived medium (**B**) with a heat map of effector molecule MFIs overlaid onto the t-SNE analysis. (**C**) t-SNE projection of CD8 + T cells for all groups with various durations and percentages of CD8 + T cells from different days in each FlowSOM cluster (n = 3). All data are mean ± SEM and were analysed by two-way ANOVA with Sidak multiple comparisons tests (**C**) with adjusted P value and data are cumulative results from at least three independent experiments. Source data

**Extended Data Fig. 6:**
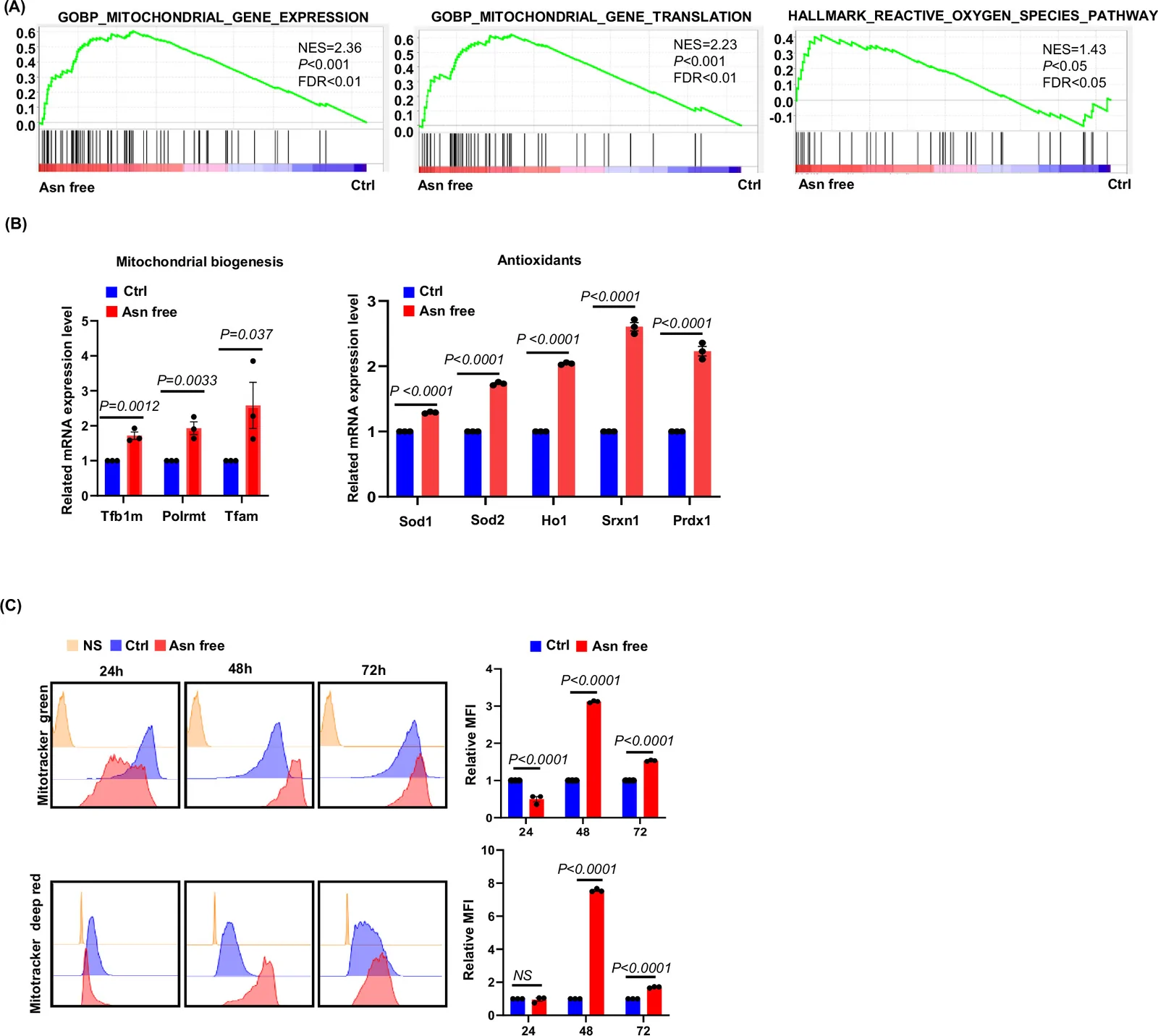
Asparagine deprivation reprograms mitochondrial fitness in T cells. (**A**) GSEA tracing for mitochondrial biogenesis in Asn-free and control cultured CD8 + T cell. ES, enrichment score; NES, normalized enrichment score; FDR, false discovery ratio. (**B**) The level of indicated mRNAs in control T cells and asparagine-free T cell were determined by qPCR (n = 3). (**C**) Naïve CD8 + T cells cultured in asparagine-free and control medium for several days. CD8 + T cells stained with MitoTracker™ Green FM Dye and MitoTracker™, Deep Red FM Dye (n = 3). All data represent mean ± SEM from three independent biological replicates. One-tailed unpaired Student’s t-test was used for individual comparisons in (**B**), while two-way ANOVA with Šídák’s multiple comparisons test was applied in (**C**). Source data

**Extended Data Fig. 7:**
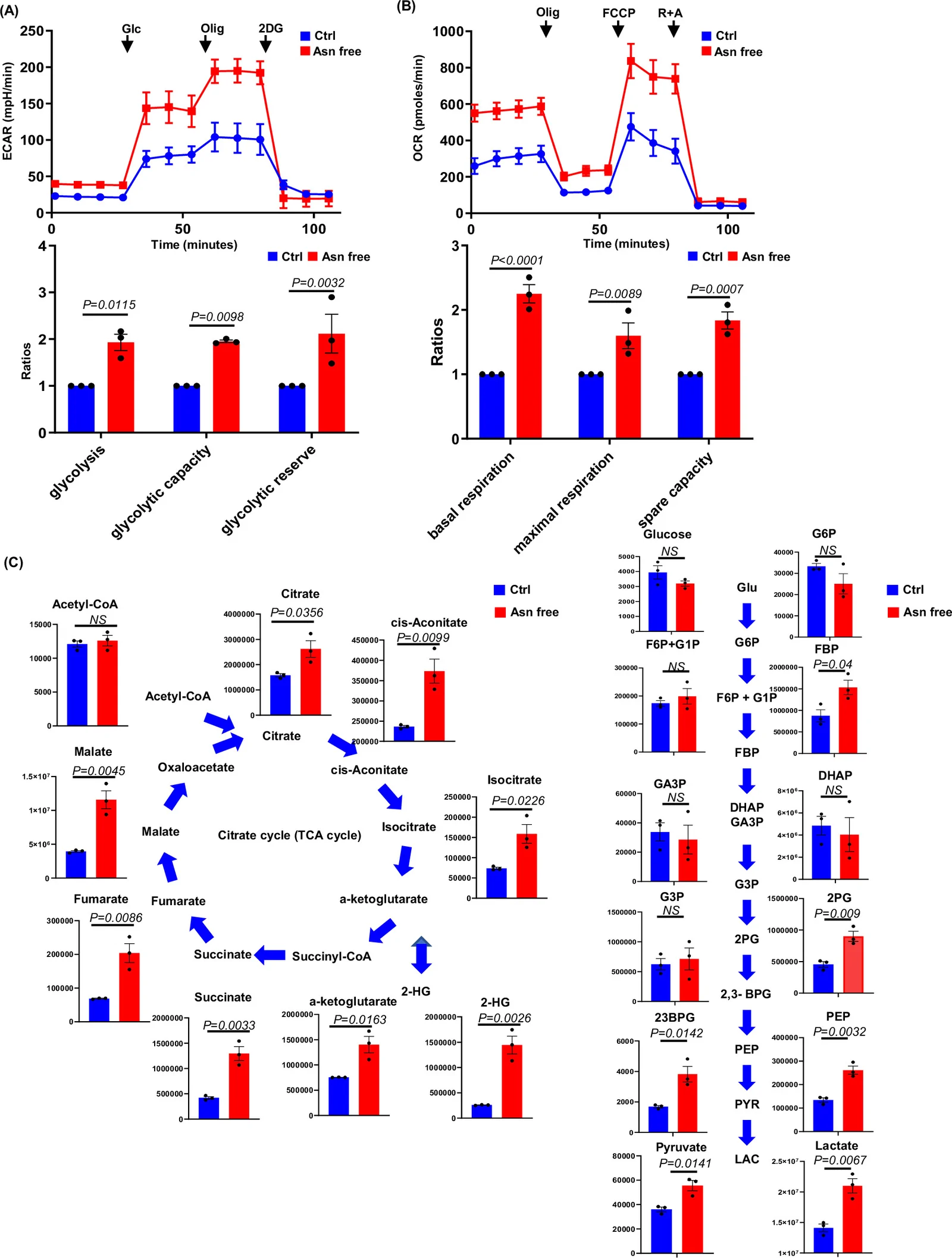
Asparagine deprivation influences metabolic preferences in T cells. (**A**) Traces of oxygen consumption rates (OCRs) were recorded (n = 3). The following compounds were injected into the assay micro-chambers as indicated: oligomycin (Olig.), carbonyl cyanide 4-(trifluoromethoxy) phenylhydrazone (FCCP), and rotenone plus antimycin A (R + A). (**B**) Traces of extracellular acidification rates (ECARs) were recorded (n = 3). Compounds injected included glucose (Glc), oligomycin, and 2-deoxy glucose (2DG). Each data point represents the mean ± standard deviation (SD) of four technical replicates. (**C**) Lysed cells from a control medium or an asparagine-deprived medium 3-day cultured CD8 T cell. The cells were subsequently analysed using liquid chromatography–mass spectrometry (LC–MS) to assess the glycolysis (right) and TCA cycle (left) (n = 3). All data represent mean ± SEM from three independent biological replicates. Two-tailed unpaired Student’s t-test was used for individual comparisons in (**C**), two-way ANOVA with Sidak multiple comparisons tests (**A**, **B**)with adjusted P value and data are cumulative results from at least three independent experiments. Source data

**Extended Data Fig. 8:**
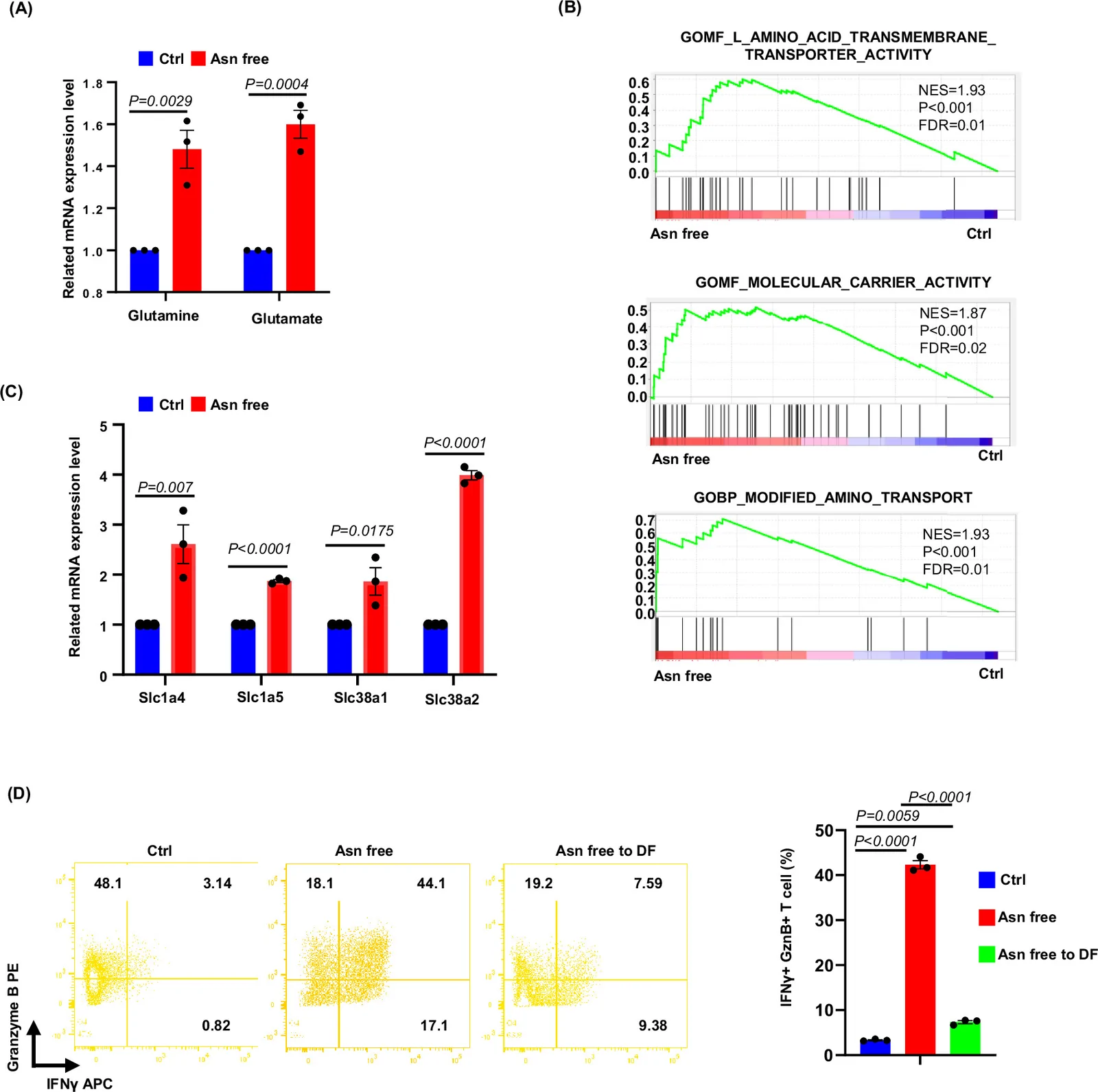
Asparagine deprivation affects glutamine metabolism to enhance CD8 T cell activation. (**A**) Lysed cells from a control medium or an asparagine-deprived medium 3-day cultured CD8 T cell. The cells were subsequently analysed using liquid chromatography–mass spectrometry (LC–MS) to assess the glutamine and glutamate (n = 3). (**B**) GSEA tracing for mitochondrial biogenesis in Asn-free and control cultured CD8 + T cell. ES, enrichment score; NES, normalized enrichment score; FDR, false discovery ratio. (**C**) The level of indicated mRNAs in control T cells and asparagine-free T cell were determined by qPCR (n = 3). (**D**) Naïve CD8 + T cells were cultured in CD8 differentiation conditions with either asparagine-free or control medium for three days. For the Asn-free to DF condition (Asparagine and Glutamine-free), after two days of culture in an asparagine-free medium, the cells were transferred to a medium lacking both asparagine and glutamine for one day. CD8 + T cells stained for IFN-γ and granzyme B and analysed by flow cytometry (n = 3). All data represent mean ± SEM from three independent biological replicates. One-tailed unpaired Student’s t-test was used for individual comparisons in (**A**, **C**), while one-way ANOVA with Tukey’s multiple comparisons test was applied in (**D**). P < 0.05 was considered statistically significant. Source data

To gain a comprehensive understanding of the impact of asparagine deprivation on T cell activation, we cultured CD8^+^ T cells in vitro in asparagine-replete and -depleted conditions over 3 days and performed FlowSOM clustering (Extended Data Fig. 5a–c). We found that asparagine deprivation could initially induce delayed T cell activation, but a potential adaptation process induced by prolonged asparagine deprivation eventually potentiates T cell differentiation towards populations with more superior effector functions. We further found that asparagine deprivation elevated gene signatures related to elevated mitochondrial activity as well as key genes related to mitochondrial biogenesis and antioxidant responses in CD8^+^ T cells (Extended Data Fig. 6a,b). Of note, acute asparagine deprivation (24 h) suppressed mitochondrial mass and membrane potential; however, there were robust increases in both parameters in T cells cultured in the asparagine-deprived condition for 48 h and 72 h (Extended Data Fig. 6c). In line with our immune profiling and a recent study^9^, our results indicate that prolonged asparagine deprivation can induce opposite outcomes in mitochondrial state as well as a T cell differentiation pattern, which potentially result from adaptation induced by prolonged asparagine deprivation. To further investigate the global impacts of asparagine deprivation on the metabolic programme, we performed a Seahorse extracellular flux assay (Extended Data Fig. 7a,b) and targeted metabolomics (Extended Data Fig. 7c). The data revealed that asparagine deprivation promoted bioenergetic programmes, including OXPHOS and glycolysis, in T cells. Notably, T cells cultured in the asparagine-depleted conditions increased glutamine and glutamate levels (Extended Data Fig. 8a) and were enriched in gene sets involved in amino acid transporter activity and glutamine transporters (Extended Data Fig. 8b,c), implying that asparagine-deprived T cells may display a potential enhancement in glutamine metabolism. To explore whether the enhancement of glutamine metabolism is required to promote effector functions in T cells upon asparagine deprivation, we activated T cells in the asparagine-replete or -depleted medium for 2 days. T cells activated in the absence of asparagine were then switched to either asparagine-depleted medium or medium deprived with both asparagine and glutamine for one more day. We found that glutamine deprivation abolished the enhancement of effector cytokine production in asparagine-deprived T cells (Extended Data Fig. 8d), indicating that glutamine plays a critical role in tailoring effector functions in response to asparagine deprivation in CD8^+^ T cells. Altogether, our results highlight that asparagine deprivation rewires metabolic programmes in a stage-dependent manner and the elevated glutamine metabolism plays a crucial role in enabling CD8^+^ T cells to mount elevated effector function in response to asparagine deprivation.

**Fig. 2:**
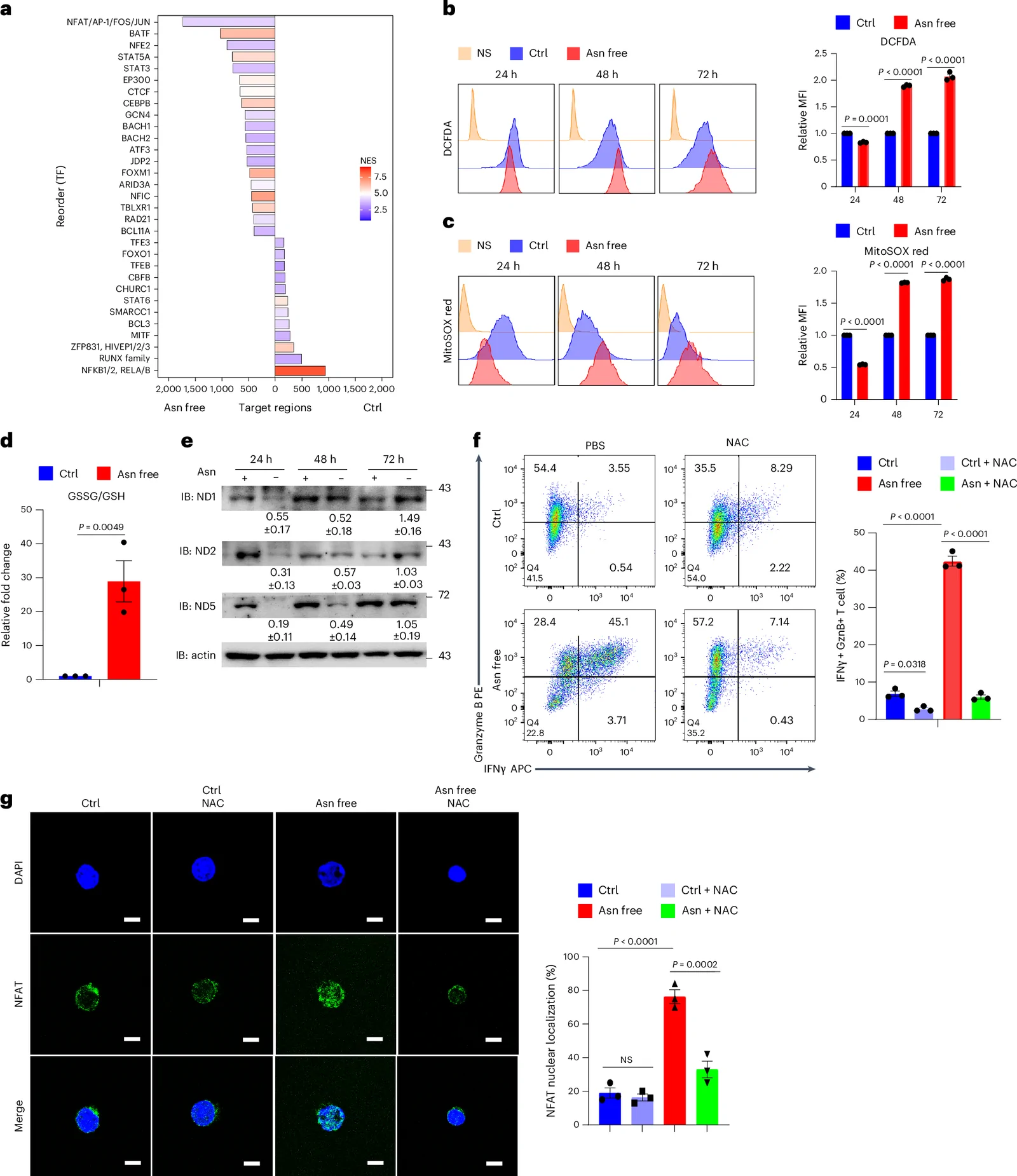
Asparagine deprivation tailors differentiation and metabolic adaptation in a ROS-dependent manner. **a**, The enriched transcription factors (TFs) in both Asn-free and control conditions are selected and the TFs that have the most target region enrichment in either Asn-free or control are represented as the preference. **b**,**c**, Naive CD8^+^T cells were cultured under CD8 differentiation conditions in both control medium and asparagine-deprived medium for various durations. Subsequently, CD8^+^T cells were stained for DCFDA (**b**) and MitoSOX (**c**) (*n* = 3). **d**, Lysed cells from a control medium or an asparagine-deprived medium 3-day cultured CD8^+^ T cell. The cells were subsequently analysed using liquid chromatography–mass spectrometry to assess the GSSG/GSH ratio (*n* = 3). **e**, Naive CD8^+^T cells were cultured under CD8^+^ differentiation conditions in both control medium and asparagine-deprived medium for various durations. The protein levels of the indicated molecules were determined through immunoblot analysis, which was independently repeated three times. Blots were cropped for clarity. Actin was run on separate gels due to different protein loading amounts. To ensure comparability, all blots were processed in parallel under identical conditions. IB, immunoblot. **f**, Naive CD8^+^T cells cultured in CD8^+^ differentiation conditions with either asparagine-free or control medium and added with 20 mM NAC for 3 days, CD8^+^ T cells stained for IFNγ and granzyme B and analysed by flow cytometry (*n* = 3). PE, phycoerythrin; APC, allophycocyanin. **g**, Imaging of NFAT localization staining (in green) and DAPI (in blue) in CD8^+^ T cells by confocal microscopy (*n* = 3). Scale bar, 5 µm. All data are mean ± s.e.m. and were analysed by one-tailed unpaired (**d**) Student’s *t*-test, one-way analysis of variance (ANOVA) with two-way ANOVA with Tukey’s multiple comparisons test (**f** and **g**) or Sidak multiple comparisons tests with adjusted *P* value (**b** and **c**), and data are cumulative results from three independent experiments. NES, normalized enrichment score. Source data

**Extended Data Fig. 9:**
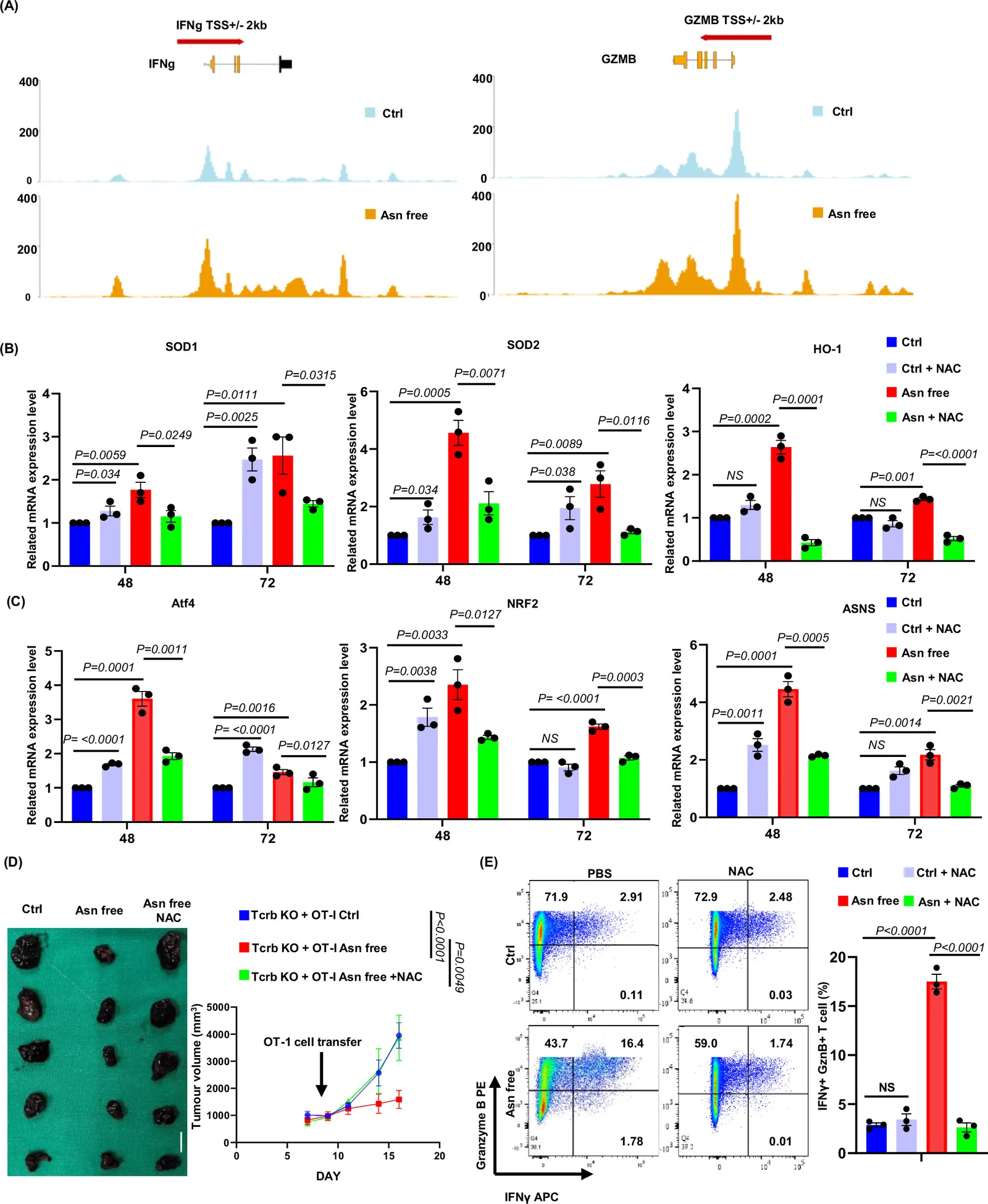
Asparagine deprivations modulates chromatin accessibility pattern in T cells. (**A**) The signal of open chromatin in both Asn-free and control conditions on Day 3 are represented in the genomics locus of IFNG and GZMB. The orange indicated the Asn-free condition whereas the blue indicated the control condition. (**B**) (**C**) The level of indicated mRNAs in T cells cultured under control and asparagine-deprived conditions in the presence of N-acetylcysteine (NAC) were determined by qPCR (n = 3). (**D**) B16-OVA-bearing Tcrb KO mice were received 1.5 × 10^6 activated OTI T cells on day 7. Data are presented as the mean ± SEM (n = 5 for each group). (**E**) human CD8 + T cells were isolated from human PBMCs using the Naive CD8 + T Cell Isolation Kit, human. CD8 + T cells cultured under control and asparagine-deprived conditions in the presence of N-acetylcysteine (NAC) for 3 days (n = 3). CD8 + T cells were stained for IFN-γ and granzyme B and analysed by flow cytometry (n = 3). All data represent mean ± SEM from three independent biological replicates. One-tailed unpaired Student’s t-test was used for individual comparisons in (**B**, **C**, **E**), while two-way ANOVA with Tukey’s multiple comparisons test was applied in (**D**). P < 0.05 was considered statistically significant. Source data

To further elucidate how asparagine deprivation modulates differentiation and effector function of CD8^+^ T cells, we performed assay for transposase-accessible chromatin with high-throughput sequencing (ATAC-seq) for CD8^+^ T cells activated in control or asparagine-depleted conditions for 3 days. We found that asparagine-deprived T cells displayed higher chromatin accessibility in gene loci, including IFNγ and GzmB, compared with the control group (Extended Data Fig. 9a). Moreover, the genomic regions that were more accessible in asparagine-deprived T cells exhibited enrichment in the consensus binding motifs for nuclear factor of activated T cells (NFAT) and basic leucine zipper transcription factor ATF-like (BATF) (Fig. 2a), two critical transcription factors controlling activation and effector differentiation of T cells^14,15^. This result highlights that asparagine deprivation may enhance NFAT-mediated transcriptional regulation to support effector T cell differentiation. As the persistent production of reactive oxygen species (ROS) has been shown to stimulate NFAT signalling^16^, and glutamine can modulate the redox balance by serving as a precursor for glutathione (GSH)^17^, we postulated that asparagine deprivation may boost ROS production and that the elevated glutamine levels may be an adaptation process to protect cells from excessive damage caused by ROS production. In support of this, we indeed observed that T cells from the asparagine-deprived condition elevated the gene signature related to the ROS pathway (Extended Data Fig. 9b). We next examined total ROS and mitochondrial ROS (mROS) in CD8^+^ T cells activated in asparagine-replete and asparagine-depleted conditions by 2′,7′-dichlorofluorescein diacetate (DCFDA) and MitoSOX, respectively. Our result showed that asparaginase deprivation initially suppressed ROS and mROS in the 24 h culture; however, prolonged deprivation boosted production of ROS and mROS after 48 h (Fig. 2b,c), which is in line with the delayed activation but enhanced effector functions as we observed in our previous results. Furthermore, we found that asparagine deprivation robustly increased glutathione disulfide (GSSG) to GSH ratios in CD8^+^ T cells (Fig. 2d), indicating that asparagine deprivation promotes production of ROS and consumption of GSH due to antioxidant responses. Notably, asparagine is a critical amino acid required for the synthesis of essential proteins and mitochondrial genome-encoded proteins, including NADH-ubiquinone oxidoreductase chain 1, 2 and 5 (ND1, ND2 and ND5)^18^. As the deficiency of ND protein subunits could lead to elevated mROS production^19^, we then examined whether asparagine deprivation could modulate the expression of ND proteins. We found that asparagine depletion robustly suppressed ND1, ND2 and ND5 protein expression (Fig. 2e). However, the expression of ND1, ND2 and ND5 was restored at 72 h after asparagine deprivation. This result suggests that the impaired expression of ND proteins in response to asparagine deprivation may support ROS production and the adaptation process induced by prolonged asparagine deprivation can restore ND protein expression. Of note, asparagine restriction has been shown to promote ROS production and activate integrated stress responses, especially activating transcription factor 4 (ATF4), in multiple cell types^20,21^. Moreover, the elevated ATF4 activity induced by asparagine restriction can stimulate the expression of asparagine synthetase (ASNS) to adapt metabolic stress^22,23^. We therefore speculated that elevated ROS can promote metabolic adaptation, including ATF4 activation, the expression of ASNS and antioxidant responses, to couple stress responses induced by asparagine restriction. To examine this, we activated T cells with a potent antioxidant, *N*-acetylcysteine (NAC), in the asparagine-replete or -depleted conditions. Our result showed that asparagine restriction induced expression of *ATF4*, *ASNS* and *NRF2* (a master transcription factor controlling oxidant signalling pathways) in an NAC-sensitive manner (Extended Data Fig. 9c), suggesting that ROS controls metabolic adaptation in response to asparagine restriction. Moreover, we found that NAC treatment abolished asparagine deprivation-mediated enhancement of effector functions in both murine and human CD8^+^ T cells (Fig. 2f and Extended Data Fig. 9d). In addition, our result showed that asparagine deprivation can promote NFAT nuclear translocation (an indication of NFAT activation)^24^, but the co-treatment of NAC impaired the enhancement of nuclear NFAT levels (Fig. 2g). Altogether, we delineated that acute asparagine restriction delayed T cell activation, but prolonged asparagine deprivation promoted effector functions in T cells. Mechanistically, prolonged asparagine deprivation led to deregulated expression of mitochondrial complex I subunits, which fostered ROS production and glutamine metabolism to tailor ROS-guided NFAT nuclear localization and activity. As a result of this metabolic adaptation induced by prolonged asparagine restriction, ROS orchestrated distinct T cell differentiation process and promoted effector function in CD8^+^ T cells. We further discovered the bi-phased regulations induced by asparagine deprivation and delineated the underlying metabolic adaptation that tailors T cell differentiation and effector functions. Most notably, our findings provide proof-of-concept evidence on exploiting asparaginase, a US Food and Drug Administration-approved chemotherapy in acute lymphoblastic leukaemia patients, with ICIs on treating patients with solid tumours.

## Methods

### Human participants

This study was a single-centre, prospective salvage therapy conducted under compassionate-use conditions, utilizing l-asparaginase for patients with refractory NPC who did not respond to standard treatment and anti-PD-1 therapy. These patients had exhausted all approved treatment regimens. Patients who visited the Oncology Department at Lin-Kou Chang Gung Memorial Hospital (CGMH) between August 2023 and March 2024 were screened. Nine patients with treatment-refractory NPC were identified, with one excluded due to an autoimmune disease (Extended Data Fig. 1a). The baseline demographic and clinical characteristics for all eight patients are presented in Table 1. Sex was self-reported at the time of screening. Eight patients were randomized to receive either combination therapy or anti-PD-1 monotherapy. In the combination therapy group, all patients received a single course of 10,000 IU m^−2^ l-asparaginase for 3 or 5 days before ongoing anti-PD-1 therapy. Apart from this single course of l-asparaginase, cancer treatment and follow-up procedures were consistent with those employed for patients with NPC at CGMH, including PET/CT follow-up, biochemical laboratory tests and EBV DNA titre monitoring. The study was approved by the Medical Ethics and Human Clinical Trial Committee of CGMH (Institutional Review Board no. 202400085B0) and complies with the ethical guidelines of ClinicalTrials.gov (NCT06676293). Written informed consent was obtained from all participants before their inclusion in the study. Patients did not receive financial compensation for their participation. Sex-disaggregated data are provided in Source Data files, and overall participant numbers are summarized in the Methods and Reporting Summary.

### Study protocol

The full study protocol, including eligibility criteria, intervention details and data collection methodology, is available upon request from the corresponding author and is also outlined in the ClinicalTrials.gov registration.

### Data collection and outcomes

Patients were monitored through routine clinical assessments, including PET/CT imaging, biochemical laboratory tests and EBV DNA titre analysis. Primary and secondary outcome measures included the assessment of tumour response to therapy, measured by PET/CT and Response Evaluation Criteria in Solid Tumours (RECIST) criteria for the primary outcome. Changes in biochemical laboratory parameters, EBV DNA levels and patient survival rates were recorded as secondary outcomes. These outcomes were predefined and analysed as described in the protocol.

### Animal studies and ethical approval

All experiments involving laboratory animals followed the guidelines for animal experiments of CGMH and were approved by the Institutional Animal Care and Use Committee (IACUC) of CGMH. The mouse strains Tcrb KO (strain 002118) and OT-1 (strain 003831) were used and obtained from The Jackson Laboratory. C57BL/6JNarl mice were purchased from the National Laboratory Animal Center (Taiwan). C57BL/6J mice were housed under standard conditions. Mice were housed in either the National Laboratory Animal Center or the conventional animal facilities at the Laboratory Animal Center, CGMH, Lin-Kou, Taiwan. They were kept in individually ventilated cages under a 12-h light–dark cycle at an ambient temperature of 19–23 °C.

### Age and sex considerations

All experiments used 6–8-week-old mice. Both male and female mice were included, with sex-matching applied across experimental groups. Sex was considered in the study design and data were analysed for potential sex-based differences where relevant.

### In vitro T cell activation studies

Naive CD8^+^T cells were isolated with naive CD8 adverse selection cocktail and LS columns (Miltenyi) from C57BL/6 wild-type mice splenocytes and lymph nodes. One million cells per ml in a 24-well plate were activated with plate-bound anti-CD3 (5 µg ml^−1^) with soluble anti-CD28 (2 µg ml^−1^) and IL-2 (10 ng ml^−1^) in control or asparagine deprivation medium. For re-stimulation, approximately 1 × 10^6^ cells per well in a 96-well plate were stimulated in the presence of GolgiStop (BD Bioscience) for 4 h at 37 °C with PMA (50 ng ml^−1^) and ionomycin (500 ng ml^−1^). Peripheral blood mononuclear cells (PBMCs) from healthy voluntary donors were isolated by density gradient centrifugation on Ficoll Paque according to the manufacturer’s instructions (Pharmacia). Human CD8^+^T cells were isolated from human PBMCs using a Naive CD8^+^T Cell Isolation kit, human (Miltenyi Biotec). One million cells per ml in a 24-well plate were activated with anti-CD3 (10 ng ml^−1^), soluble anti-CD28 (2 µg ml^−1^) and IL-2 (40 U ml^−1^; Genzyme), plate-bound anti-CD3 (5 µg ml^−1^) with soluble anti-CD28 (2 µg ml^−1^) and IL-2 (10 ng ml^−1^) in control or asparagine deprivation medium.

### Tumour growth and survival experiments

For combination l-asparaginase and immune-checkpoint experiments, l-asparaginase and anti-PD-1 mAb (RMP1-14, Bio X Cell) were used in therapy experiments. Tumour injections were administered on the right flank. C57BL/6 wild-type mice were injected subcutaneously (s.c.) with 5 × 10^5^ for B16-F10 or 1 × 10^6^ for MTCQ1 tumour cells cultured in a DMEM-based medium. l-asparaginase was dissolved in PBS and administered for all vehicle-treated control experiments. Groups were randomized based on tumour size on the day of beginning treatment. Mice were treated with l-asparaginase (5 U g^−1^) or anti-PD-1 mAb (RMP1-14, Bio X Cell) was administered by intraperitoneal (i.p.) injection (100 μg per mouse) for four doses on days 10, 12, 14 and 16. For adoptive cellular therapy experiments, C57BL/6 wild-type mice received a s.c. injection of 5 × 10^5^ B16-OVA-Luc melanoma cells cultured under OVA selection medium containing 400 µg ml^−1^ G418 (Life Technologies). Mice were treated with l-asparaginase or vehicle three times weekly in 100 μl for days 7 and 9 post-tumour inoculation. Ten days after tumour injection, mice received an adoptive transfer of 1.5 × 10^6^ activated OTI (OT-I (ovalbumin-specific T cell receptor transgenic) mice) cells via intravenous injection. Mice were randomized based on tumour size before initiating combination therapy or transferring activated OTI cells in the case of adoptive transfer experiments. Tumour burden was assessed every 2–4 days by measuring the length and width of the tumour. Tumour volume was calculated using the formula *V* = (*L* × *W* × *W*)/2, where *V* is tumour volume, *W* is tumour width and *L* is tumour length. For IVIS Imaging, luciferin, the substrate for luciferase, was administered i.p. at a dose of 100 mg kg^−1^ body weight. After injection, mice were anaesthetized and positioned on the imaging stage of the IVIS apparatus in the abdominal (s.c. model) position. Images were acquired every few minutes from 10 to 30 min post-injection using the IVIS Spectrum 3D system. Photon emission from the tumour and surrounding tissues was quantified using Living Image Software (Aura Imaging Software v.4.0). Mice were killed when tumours reached 2 cm in any dimension, became ulcerate or necrotic, or caused functional deficits. Unless otherwise specified, the mean tumour volume is depicted until the time of first killing. All experiments involving laboratory animals followed the guidelines for animal experiments of CGMH and were approved by the IACUC of CGMH.

### Tumour-infiltrating lymphocyte isolation

Tumours were collected from killed mice on day 18 post-tumour inoculation. Isolated tumours were incubated in collagenase type I (Gibco) and DNase (Roche) in RPMI for 30 min at 37 °C. Tumour mixtures and spleens were dissociated through a 70-µm filter and washed with PBS. Tumour suspensions were pelleted and resuspended in 40% Percoll solution, underlaid with 80% Percoll in a 15-ml conical flask. After centrifugation at 2,000*g* for 30 min, the middle layer was removed, washed in PBS and counted. For in vitro stimulation assays, approximately 1 × 10^6^ cells per well in a 96-well plate were stimulated in the presence of GolgiStop (BD Bioscience) for 4 h at 37 °C with PMA (50 ng ml^−1^) and ionomycin (500 ng ml^−1^).

### FACS analysis and antibodies

The following clones of antibody were purchased from BioLegend or BD Bioscience and used for cell surface staining. For the human panel: LAG-3 (11C3C65, 1:200 dilution), CD279 (EH12.1, 1:200 dilution), CD8 (RPA-T8, 1:200 dilution), ICOS (DX29, 1:100 dilution), CD4 (SK3, 1:200 dilution), Tim-3 (7D3, 1:100 dilution), CD3 (UCHT1, 1:200 dilution), CD152 (BNI3, 1:100 dilution), CD45RA (HI100, 1:200 dilution), KLRG1 (13F12F2, 1:100 dilution), CD69 (FN50, 1:200 dilution), CD127 (A019D5, 1:100 dilution), CD25 (2A3, 1:200 dilution), TIGIT (A15153G, 1:200 dilution), CD197 (150503, 1:100 dilution) and CD45RO (UCHL1, 1:200 dilution). For the mouse panel: CD25 (PC61, 1:200 dilution), TCRβ (H57-597, 1:200 dilution), TIGIT (1G9, 1:200 dilution), CD366 (5D12, 1:200 dilution), Ly6C (HK1.4, 1:200 dilution), CD44 (IM7, 1:200 dilution), CD223 (C9B7W, 1:200 dilution), CD279 (29F.1A12, 1:200 dilution), CD152 (UC10-4B9, 1:200 dilution), KLRG1 (2F1, 1:200 dilution), CD62L (MEL-14, 1:200 dilution) and CD127 (A7R34, 1:100 dilution). For nuclear protein staining, the cells were fixated and permeabilized using the FOXP3 Transcription Factor Staining kit (Thermo Fisher Scientific) according to the manufacturer’s instructions. For human samples: IFNγ (B27, 1:400 dilution), IL-2 (MQ1-17H12, 1:200 dilution), Granzyme B (GB11, 1:400 dilution), TCF-7 (S33-966, 1:200 dilution), TOX (TXRX10, 1:200 dilution), T-bet (4B10, 1:400 dilution) and TNF (MAb11, 1:400 dilution). For mouse samples: TNF (MP6-XT22, 1:400 dilution), RORγt (Q31-378, 1:200 dilution), CD45 (30-F12, 1:200 dilution) and TCF-7 (S33-966, 1:200 dilution). Flow cytometry was performed on a BD FACSymphony A5 Cell Analyzer or BD FACSVerse Cell Analyzer and analysed with FlowJo software (v.10.8.1).

### Western blot analysis

Total protein was extracted as previously described^25^ and resolved using 10% SDS–PAGE. Proteins were then transferred onto PVDF membranes (Merck Millipore) via electroblotting. To block nonspecific binding, membranes were incubated with 5% nonfat milk in TBST (Tris-buffered saline with 0.1% Tween-20) for 1 h at room temperature. Membranes were incubated overnight at 4 °C with primary antibodies at the following dilutions: ND1 (Proteintech, cat. no. 19703-1-AP, 1:500 dilution), ND2 (Proteintech, cat. no. 19704-1-AP, 1:500 dilution), MT-ND5 (Proteintech, cat. no. 55410-1-AP, 1:500 dilution), NDUFA5 (Proteintech, cat. no. 16640-1-AP, 1:500 dilution) and β-actin (1:20,000 dilution used as a loading control). After primary antibody incubation, membranes were washed with TBST and incubated with horseradish peroxidase (HRP)-conjugated secondary antibodies for 1 h at room temperature. Immunoreactive bands were detected using an enhanced chemiluminescence detection system (Amersham). Band intensities were quantified by scanning densitometry and normalized to β-actin levels.

### Immunofluorescence

Mouse CD8^+^T cells were stimulated with plate-bound anti-CD3 and anti-CD28 antibodies. After stimulation, cells were resuspended in fresh medium and seeded onto poly-l-lysine-coated coverslips in six-well culture plates. Cells were incubated at 37 °C for 1 h to allow adherence. Cells were then fixed and permeabilized using the FOXP3 Transcription Factor Staining kit (Thermo Fisher Scientific) according to the manufacturer’s instructions. After permeabilization, cells were blocked with cold blocking buffer (1× PBS, 0.5% standard-grade BSA) for 30 min on ice. Following blocking, the buffer was carefully removed and cells were incubated overnight at 4 °C with NFAT1 (D43B1) XP Rabbit mAb (5861, Cell Signalling Technology) at 1:100 dilution. The next day, cells were incubated with Alexa Fluor 488-conjugated secondary antibody (Molecular Probes) for 1 h on ice in the dark, followed by three PBS washes. For nuclear staining, cells were incubated with 4′,6-diamidino-2-phenylindole (DAPI) (Thermo Fisher Scientific). Finally, immunofluorescent images were captured using a Leica TCS SP8 X confocal microscope (Leica Microsystems).

### Metabolic assays

For Seahorse metabolic flux analyses, isolated CD8^+^T cells were activated for 3 days (as described above), washed and plated on Seahorse metabolic flux analysis plates at 5 × 10^4^ cells per well in a 24-well container. Experiments were conducted in an XF assay medium that contained 25 mM glucose, 2 mM l-glutamine and 1 mM sodium pyruvate and analysed using a Seahorse XF24 extracellular flux analyzer (Agilent Technologies). When indicated, the following were injected: oligomycin (1.5 μM), carbonyl cyanide 4-(trifluoromethoxy) phenylhydrazone (FCCP; 1.5 μM), rotenone (100 nM) and antimycin A (1 μM). Basal extracellular acidification rate and oxygen consumption rate reports were generated by Seahorse Wave Desktop Software (v.2.2.0.276)

### Total RNA extraction and RT–qPCR

Total RNA was extracted from cells using TRIzol reagent (Invitrogen) or extracted using EasyPrep Total RNA kit (BIOTOOLS, DPT-BD19) following the manufacturer’s instructions. The quantity and quality of extracted RNA were verified using a NanoDrop spectrophotometer (Thermo Fisher Scientific) and Agilent 2100 Bioanalyzer (Agilent Technologies). Quantitative PCR with reverse transcription (RT–qPCR) was performed using a first-strand complementary DNA synthesis kit for RT–PCR (Roche Diagnostics), and an ABI ViiA7 real-time PCR system (Applied Biosystems) using TaqMan gene expression assays. The results are expressed as a threshold cycle (*Ct*). The expression level of each gene was normalized to that of GAPDH, and the relative messenger RNA expression data were calculated using the 2^-ΔΔ*Ct*^ method. All experimental samples were compared with the control (uninfected control groups) and expressed as an *n*-fold difference. All primers used in this study were purchased from Thermo Fisher Scientific. The assay ID is provided in Supplementary Data. The RT–PCR data were statistically analysed by determining the fold change values. We performed all statistical analyses using GraphPad Prism (v.9) and R software (v.4.3.3). For comparisons between two groups, we used unpaired two-tailed *t*-tests. For multiple group comparisons, one-way analysis of variance (ANOVA) or Kruskal–Wallis tests were performed, followed by appropriate post hoc analysis.

### Gene set enrichment analysis

Gene set enrichment analysis (GSEA; http://www.broad.mit.edu/GSEA) was performed to explore the biological pathways involved in glutamine deprivation. We used the built-in C2, C5 and C6 curated gene sets from Molecular Signatures Database (MSigDB; v.6.0; www.broadinstitute.org/gsea/msigdb). The statistical significance of GSEA was analysed using 1,000 permutations. Enrichment was compared between control and asparagine-deprivation CD8^+^T cells. A positive enrichment score indicates that the specific molecular signature correlated with the phenotype of control CD8^+^ cells. The resulting pathways are selected using the normalized enrichment score >1 and nominal *P* value < 0.05.

### RNA-sequencing

Total RNA was isolated from in vitro cultured CD8^+^T cells using TRIzol reagent (Invitrogen), according to the manufacturer’s protocol. A NanoDrop spectrophotometer (Thermo Fisher Scientific) and an Agilent 2100 Bioanalyzer (Agilent Technologies) were used to evaluate the quantity and integrity of the extracted RNA. A paired-end sequencing approach was used to sequence the samples on an Illumina sequencing platform (Illumina). The sequencing data were then mapped to the reference genome (*Mus* *musculus*; GRCm38) using CLC Genomics Workbench v.9.5 software (CLC bio). The reads per kilobase million number was used to calculate gene expression levels. To identify and compare pathways enriched by the differentially expressed genes from the RNA-seq datasets.

### ATAC-seq

The ATAC-Seq kit (cat. no. 53150, Active Motif North America) was employed for tagmentation and library preparation following the kit procedures. In brief, T cells were activated and cultured in standard (Con) or asparagine-free (Asn) medium for 3 days, and 50,000 to 100,000 cells were collected and lysed with 100 μl ice-cold ATAC Lysis Buffer. Subsequently, tagmentation and library preparation were performed according to the instructions of the kit. Three biological replicates were conducted. The sequencing was performed on NovaSeq 6000 with paired-end 150 bp. Raw reads were trimmed by using NGmerge (v.0.3) and aligned to the mm10 using Bowtie2 (v.2.2.4). The peak calling as well as the removal of duplicate reads and mitochondria chromosome were performed using Genrich (v.0.6.1). The identified peaks from all samples were merged into a consensus peak list, and the chromatin accessibility of each consensus peak in each sample was quantified by using featureCounts function in R with Rsubread package. The presences of open chromatin were used to perform PCA by R. The differential accessible regions were calculated by R with the DESeq2 package. The differentially accessible regions were defined according to *P* ≤ 0.05 and |log_2_(fold change)| ≥1 for the following transcription factor enrichment analysis. The transcription factor enrichment analysis was performed by i-cisTarget^26,27^.

### ^18^F-FDG PET/CT imaging

All enrolled patients adhered to a minimum fasting duration of 6 h, in brief, undergoing ^18^F-FDG PET/CT imaging, utilizing a PET/CT system (Discovery ST 16; GE Healthcare) comprising a PET scanner and a 16-section CT scanner. Before PET acquisition, a helical CT scan was performed from the head to the proximal thigh following a standardized protocol with the following parameters: transverse 3.0-mm collimation × 16 modes, 100 kVp, 100 mAs, tube rotation: 0.5-s tube rotation, table speed: 35 mm s^−1^, and pitch: 1.5. Intravenous iodinated contrast agents were not administered. The CT data were resized from a 512 × 512 matrix to a 128 × 128 matrix to align with PET data, facilitating the fusion of corresponding images and the generation of CT-based transmission maps. Emission scans, covering the region from the head to the proximal thigh, were conducted 50–70 min after the injection of 370 MBq of ^18^F-FDG. Two-dimensional mode acquisition (3 min for each table position) was employed. PET images underwent reconstruction using CT data for attenuation correction, utilizing an ordered-subset expectation maximization iterative reconstruction algorithm (four iterations and ten subsets). A nuclear medicine physician reviewed all images and identified lesions characterized by an increased tracer uptake. A General Electric (GE) Advanced Workstation Server software (GE Healthcare) was used for manual delineation of volumes of interest on each lesion. The segmentation of lesions was performed by applying a threshold for voxel SUVs as 3 g ml^−1^. We calculated total lesion glycolysis, maximum SUV (SUVmax), peak SUV and metabolic tumour volume in line with previous studies^28,29^.

### Determination of EBV DNA copy number

The EBV DNA copy number for each patient was determined following previously established procedures^30,31^. In brief, plasma samples were acquired by centrifuging venous blood collected in an EDTA-coated tube. The cell-free DNA was extracted using a QIAamp DNA Blood Mini kit (QIAGEN). The EBV DNA copy number was quantified using a real-time qPCR system targeting the BamHI-W fragment region of the EBV genome, employing the Applied Biosystems 7500 qPCR system (Thermo Fisher Scientific). The plasma concentration of EBV DNA (copies per ml) was calculated after calibration with the EBV World Health Organization international standard.

### Statistical methods

No statistical methods were used to pre-determine sample sizes; however, our sample sizes are similar to those reported in previous publications^9^. Statistical analysis was performed using GraphPad Prism v.9. The data distribution was assumed to be normal but this was not formally tested. Statistical tests were chosen based on the assumptions of normality and equal variances. In cases where normality could not be formally tested, the individual data points are presented in the figures for reference.

### Randomization

For all mouse experiments, groups were randomized and assigned based on genotype, sex and weight to ensure balance across experimental conditions. For experiments not involving mice, samples were allocated into experimental groups based on predefined conditions, such as cell passage number, culture duration and treatment exposure time, to maintain consistency and reproducibility across experiments.

### Blinding

Investigators were not blinded during the experiments. Blinding was not feasible because mouse genotypes and cell characteristics required screening before group allocation. However, efforts were made to minimize bias by ensuring consistent experimental conditions and objective data analysis methods.

### Data exclusion

No data were excluded from the analyses. All data collected during the study were included in the final analyses.

### Reporting summary

Further information on research design is available in the Nature Portfolio Reporting Summary linked to this article.

## Supplementary information

Supplementary Information FACS gating strategy.

Reporting Summary

Supplementary Table qPCR primer list.

## Source data

Source Data Fig. 2 Western blot raw data.

Source Data Fig. 2 Western blot raw data.

Source Data Fig. 2 Western blot raw data.

Source Data Fig. 2 Western blot raw data.

Source Data Fig. 1 Statistical source data.

Source Data Fig. 2 Statistical source data.

Source Data Extended Data Fig. 1 Statistical source data.

Source Data Extended Data Fig. 2 Statistical source data.

Source Data Extended Data Fig. 3 Statistical source data.

Source Data Extended Data Fig. 4 Statistical source data.

Source Data Extended Data Fig. 5 Statistical source data.

Source Data Extended Data Fig. 6 Statistical source data.

Source Data Extended Data Fig. 7 Statistical source data.

Source Data Extended Data Fig. 8 Statistical source data.

Source Data Extended Data Fig. 9 Statistical source data.

## Data Availability

The sequencing data generated in this study have been deposited in the NCBI Sequence Read Archive (SRA) under the following BioProject accession numbers: PRJNA1219535 (RNA-seq) and PRJNA1219369 (ATAC-seq). These datasets include RNA-seq and ATAC-seq data, which are publicly available. The corresponding metadata files and SRA accessions can be downloaded from the NCBI SRA database. Clinical data supporting the findings of this study are available upon request by contacting the corresponding author (H.-Y.Y.). All other data supporting the findings of this study are available within the paper and its Supplementary Information files. Source data are provided with this paper.
