# Author Correction: Asparagine deprivation enhances T cell antitumour response in patients via ROS-mediated metabolic and signal adaptations

**DOI:** 10.1038/s42255-026-01633-6

**Published:** 2026-09-15

**Authors:** Hsuan-Chia Chang, Chung-Ying Tsai, Cheng-Lung Hsu, Tzong-Shyuan Tai, Mei-Ling Cheng, Yu-Ming Chuang, Hsiang-Yu Tang, Kun-Ju Lin, Jia-Jin Chen, Szu-Han Chang, Yi-Ching Ko, Yu-Wen Chi, Hsuan Liu, Bertrand Chin-Ming Tan, Chia-Rui Shen, Chih-Wei Yang, Ping-Chih Ho, Huang-Yu Yang

**Affiliations:** 1 Graduate Institute of Biomedical Sciences, College of Medicine, Chang Gung University, Taoyuan, Taiwan; 2 Kidney Research Center and Department of Nephrology, Chang Gung Memorial Hospital, College of Medicine, Chang Gung University, Taoyuan, Taiwan; 3 Division of Hematology-Oncology, Department of Internal Medicine, Chang Gung Memorial Hospital, Chang Gung University, Taoyuan, Taiwan; 4 School of Medicine, Chang Gung University, Taoyuan, Taiwan; 5 Advanced Immunology Laboratory, Chang Gung Memorial Hospital, Taoyuan, Taiwan; 6 Department of Biomedical Sciences, College of Medicine, Chang Gung University, Taoyuan, Taiwan; 7 Clinical Metabolomics Core Laboratory, Chang Gung Memorial Hospital, Taoyuan, Taiwan; 8 Metabolomics Core Laboratory, Healthy Aging Research Center, Chang Gung University, Taoyuan, Taiwan; 9 Department of Oncology, University of Lausanne, Lausanne, Switzerland; 10 Ludwig Institute for Cancer Research, University of Lausanne, Epalinges, Switzerland; 11 Healthy Aging Research Center, Department of Medical Imaging and Radiological Sciences, College of Medicine, Chang Gung University, Taoyuan, Taiwan; 12 Department of Nuclear Medicine and Molecular Imaging Center, Chang Gung Memorial Hospital, Taoyuan, Taiwan; 13 Molecular Medicine Research Center, Chang Gung University, Taoyuan, Taiwan; 14 Department of Cell and Molecular Biology, College of Medicine, Chang Gung University, Taoyuan, Taiwan; 15 Division of Colon and Rectal Surgery, Lin-Kou Medical Center, Chang Gung Memorial Hospital, Taoyuan, Taiwan; 16 Research Center for Emerging Viral Infections, Chang Gung University, Taoyuan, Taiwan; 17 Department of Neurosurgery, Lin-Kou Medical Center, Chang Gung Memorial Hospital, Taoyuan, Taiwan; 18 PhD Program in Biotechnology Industry, College of Medicine, Chang Gung University, Taoyuan, Taiwan; 19 Center of Molecular and Clinical Immunology, Chang Gung University, Taoyuan, Taiwan; 20 Department of Ophthalmology, Linkou Chang Gung Memorial Hospital, Taoyuan, Taiwan; 21 Department of Health Policy and Management, Johns Hopkins Bloomberg School of Public Health, Baltimore, MD USA

**Keywords:** Immunology, Cancer therapy, Metabolism

Correction to: *Nature Metabolism* https://doi.org/10.1038/s42255-025-01245-6, published online 5 March 2025.

In the version of the article initially published, there was a copy–paste error in the Extended Data Fig. 5c source data. This has now been corrected, and Extended Data Fig. 5c has been updated in the HTML and PDF versions of the article. An original, uncorrected version of the figure is available for comparison in the Supplementary Information accompanying the amendment.

## Supplementary information

Original, uncorrected Extended Data Fig. 5

