# Supplementary material for: Asparagine deprivation enhances T cell antitumour response in patients via ROS-mediated metabolic and signal adaptations
Source: Nat Metab. 2025 Mar 5;7(5):918–27. doi: 10.1038/s42255-025-01245-6 (PMC12116382; doi:10.1038/s42255-025-01245-6)
Supplement: Supplementary file 1 — FACS gating strategy. [file 42255_2025_1245_MOESM1_ESM.pdf]

nature metabolism

Supplementary information https://doi.org/10.1038/s42255-025-01245-6

Asparagine deprivation enhances T cell antitumour response in patients via ROS-mediated metabolic and signal adaptations

In the format provided by the authors and unedited

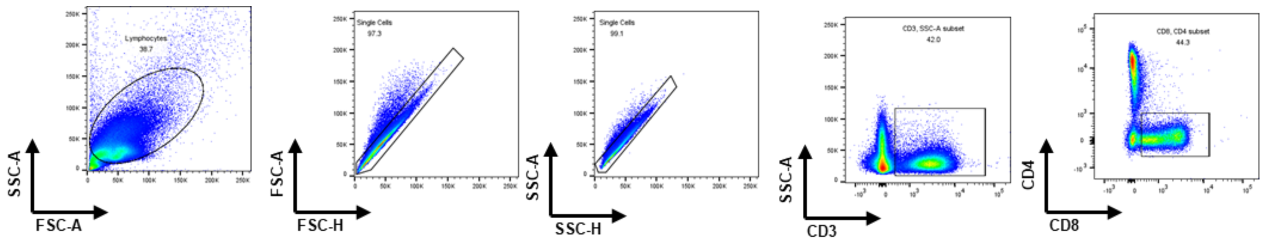

Supplemental Figure 1. Gating strategy for analysis of Figure 1B. FSC-SSC-H gating was used as preliminary gating for the lymphocyte population. (1) Lymphocytes were selected based on FSC-A vs SSC-A, (2) Single cells were identified using FSC-H vs FSC-A, (3) CD3+ cells were gated, (4) CD8+ T cells were isolated for further analysis.

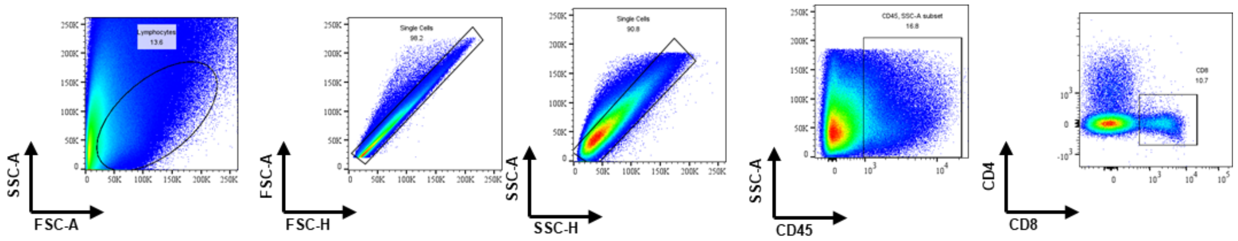

Supplemental Figure 2. Gating strategy for analysis of **Extending Figure 3A**. FSC-SSC-H gating was used as preliminary gating for the lymphocyte population. (1) Lymphocytes were selected based on FSC-A vs SSC-A, (2) Single cells were identified using FSC-H vs FSC-A, (3) CD45+ cells were gated, (4) CD8+ T cells were isolated for further analysis.

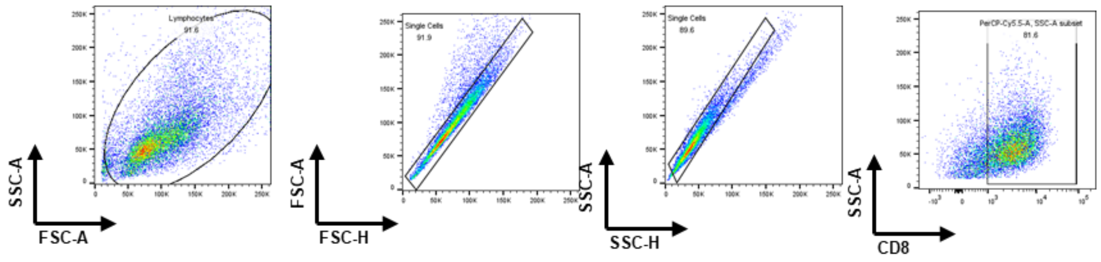

Supplemental Figure 3. Gating strategy for analysis of in vitro mouse CD8+ T cell activation. FSC-SSC-H gating was used as preliminary gating for the lymphocyte population. (1) Lymphocytes were selected based on FSC-A vs SSC-A, (2) Single cells were identified using FSC-H vs FSC-A, (3) CD8+ T cells were isolated for further analysis.

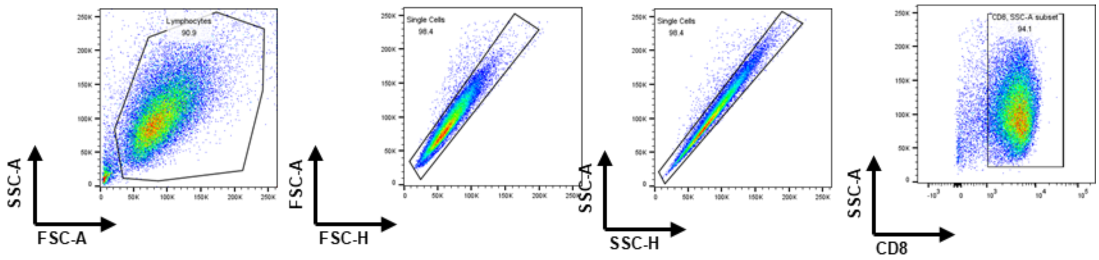

Supplemental Figure 4. Gating strategy for analysis of in vitro human CD8+ T cell activation. FSC-SSC-H gating was used as preliminary gating for the lymphocyte population. (1) Lymphocytes were selected based on FSC-A vs SSC-A, (2) Single cells were identified using FSC-H vs FSC-A, (3) CD8+ T cells were isolated for further analysis.
