# Supplementary material for: Asparagine deprivation enhances T cell antitumour response in patients via ROS-mediated metabolic and signal adaptations
Source: Nat Metab. 2025 Mar 5;7(5):918–27. doi: 10.1038/s42255-025-01245-6 (PMC12116382; doi:10.1038/s42255-025-01245-6)
Supplement: Supplementary file 2 — Reporting Summary [file 42255_2025_1245_MOESM2_ESM.pdf]

nature portfolio

Corresponding author(s): Ping-Chih Ho and Huang-Yu Yang

Last updated by author(s): Feb 5, 2025

Reporting Summary

Nature Portfolio wishes to improve the reproducibility of the work that we publish. This form provides structure for consistency and transparency in reporting. For further information on Nature Portfolio policies, see our Editorial Policies and the Editorial Policy Checklist.

Statistics

For all statistical analyses, confirm that the following items are present in the figure legend, table legend, main text, or Methods section.

n/a	Confirmed	
☐	☒	The exact sample size (*n*) for each experimental group/condition, given as a discrete number and unit of measurement
☐	☒	A statement on whether measurements were taken from distinct samples or whether the same sample was measured repeatedly
☐	☒	The statistical test(s) used AND whether they are one- or two-sided<br>*Only common tests should be described solely by name; describe more complex techniques in the Methods section.*
☒	☐	A description of all covariates tested
☐	☒	A description of any assumptions or corrections, such as tests of normality and adjustment for multiple comparisons
☐	☒	A full description of the statistical parameters including central tendency (e.g. means) or other basic estimates (e.g. regression coefficient) AND variation (e.g. standard deviation) or associated estimates of uncertainty (e.g. confidence intervals)
☐	☒	For null hypothesis testing, the test statistic (e.g. *F*, *t*, *r*) with confidence intervals, effect sizes, degrees of freedom and *P* value noted<br>*Give P values as exact values whenever suitable.*
☒	☐	For Bayesian analysis, information on the choice of priors and Markov chain Monte Carlo settings
☒	☐	For hierarchical and complex designs, identification of the appropriate level for tests and full reporting of outcomes
☒	☐	Estimates of effect sizes (e.g. Cohen's *d*, Pearson's *r*), indicating how they were calculated

Our web collection on statistics for biologists contains articles on many of the points above.

Software and code

Policy information about availability of computer code

Data collection	BD FACSVerse™ Cell Analyzer (BD Biosciences)<br>BD FACSymphony™ A5 Cell Analyzer (BD Biosciences)<br>IVIS® Spectrum 3D system(Spectral Instruments Imaging)<br>Seahorse XFe24 Analyzer(Agilent)<br>ViiA 7 Real-Time PCR System(thermofisher)<br>Applied Biosystems 7500 qPCR system (Thermo Fisher Scientific)<br>PET/CT system (Discovery ST 16; GE Healthcare, Milwaukee, WI)<br>NanoDrop spectrophotometer (Thermo Fisher Scientific, Waltham, MA, USA)<br>Agilent 2100 Bioanalyzer (Agilent Technologies, Santa Clara, CA, USA)<br>Illumina sequencing platform (Illumina, San Diego, CA, USA)<br>Leica TCS SP8 X confocal microscope (Leica Microsystems).
Data analysis	FlowJo™ v10.8.1 Software<br>Graphpad Prism 9 Software<br>Aura Imaging Software v 4.0<br>Seahorse Wave Desktop Software(2.2.0.276)<br>General Electric (GE) Advanced Workstation (AW) Server software (GE Healthcare, Waukesha, WI, USA)<br>R software (version 4.3.3)<br>CLC Genomics Workbench v9.5 software (CLC bio, Aarhus, Denmark)<br>NGmerge (v.0.3)

Bowtie2 (v.2.2.4)
Genrich (v.0.6.1)

For manuscripts utilizing custom algorithms or software that are central to the research but not yet described in published literature, software must be made available to editors and reviewers. We strongly encourage code deposition in a community repository (e.g. GitHub). See the Nature Portfolio guidelines for submitting code & software for further information.

Data

Policy information about availability of data

All manuscripts must include a data availability statement. This statement should provide the following information, where applicable:

- Accession codes, unique identifiers, or web links for publicly available datasets
- A description of any restrictions on data availability
- For clinical datasets or third party data, please ensure that the statement adheres to our policy

The sequencing data generated in this study have been deposited in the NCBI Sequence Read Archive (SRA) under the following BioProject accession numbers: PRJNA1219535 (RNA-seq) and PRJNA1219369 (ATAC-seq). These datasets include RNA-seq and ATAC-seq data, which are publicly available. The corresponding metadata files and SRA accessions can be downloaded from the NCBI SRA database. Clinical data supporting the findings of this study are available upon request by contacting the corresponding author (Professor Huang-Yu Yang). All other data supporting the findings of this study are available within the paper and its supplementary information files. Source data are provided with this paper.

Research involving human participants, their data, or biological material

Policy information about studies with human participants or human data. See also policy information about sex, gender (identity/presentation), and sexual orientation and race, ethnicity and racism.

Reporting on sex and gender	Sex was self-reported by participants, and both male and female patients were included in the study.
Reporting on race, ethnicity, or other socially relevant groupings	Race was collected through self-reporting. All participants in this study were of Chinese ethnicity, as the study was conducted in a predominantly Chinese population.
Population characteristics	This study focused on patients with treatment-refractory nasopharyngeal carcinoma (NPC). The baseline demographic and clinical characteristics of the eight participants are detailed in Table 2. Participants included individuals aged 35–65 years, with diagnoses confirmed based on clinical and pathological criteria.
Recruitment	Patients who visited the Oncology Department at Linkou Chang Gung Memorial Hospital between August 2023 and March 2024 were screened. Nine patients were screened, one of whom was excluded due to autoimmune disease, leaving eight patients who were randomized to receive either combination therapy (L-asparaginase and anti-PD1) or anti-PD1 monotherapy. Recruitment was unbiased, and inclusion criteria ensured the enrollment of eligible candidates only.
Ethics oversight	The study was approved by the Medical Ethics and Human Clinical Trial Committee of Chang Gung Memorial Hospital (IRB No. 202400085B0) and complies with the ethical guidelines of ClinicalTrials.gov (ID: NCT06676293). Written informed consent was obtained from all participants prior to their inclusion in the study.

Note that full information on the approval of the study protocol must also be provided in the manuscript.

Field-specific reporting

Please select the one below that is the best fit for your research. If you are not sure, read the appropriate sections before making your selection.

☒ Life sciences ☐ Behavioural & social sciences ☐ Ecological, evolutionary & environmental sciences

For a reference copy of the document with all sections, see nature.com/documents/nr-reporting-summary-flat.pdf

Life sciences study design

All studies must disclose on these points even when the disclosure is negative.

Sample size	The exact sample sizes for all in vivo experiments are detailed in the figure legends. No statistical methods were used to pre-determine sample sizes; however, the chosen sample sizes align with those reported in previous studies (ref 9.31) and were determined based on experimental feasibility and expected effect sizes. All in vitro experiments included at least three biological replicates per group. Statistical methods were not used to pre-determine sample sizes, as the selected sizes were deemed sufficient based on prior studies and experimental feasibility.
Data exclusions	No data were excluded from the analyses. All data collected during the study were included in the final analyses.
Replication	All experiments were performed with biological replicates, and the consistency of results across replicates is described in the figure legends.
Randomization	For all mouse experiments, groups were randomized and assigned based on genotype, sex, and weight to ensure balance across experimental

Randomization	conditions. For experiments not involving mice, samples were allocated into experimental groups based on predefined conditions, such as cell passage number, culture duration, and treatment exposure time, to maintain consistency and reproducibility across experiments.
Blinding	Investigators were not blinded during the experiments. Blinding was not feasible as mouse genotypes and cell characteristics required screening before group allocation.

Reporting for specific materials, systems and methods

We require information from authors about some types of materials, experimental systems and methods used in many studies. Here, indicate whether each material, system or method listed is relevant to your study. If you are not sure if a list item applies to your research, read the appropriate section before selecting a response.

Materials & experimental systems

n/a	Involved in the study	
☐	☒	Antibodies
☐	☒	Eukaryotic cell lines
☒	☐	Palaeontology and archaeology
☐	☒	Animals and other organisms
☐	☒	Clinical data
☒	☐	Dual use research of concern
☒	☐	Plants

Methods

n/a	Involved in the study	
☒	☐	ChIP-seq
☐	☒	Flow cytometry
☒	☐	MRI-based neuroimaging

Antibodies

Antibodies used

For the human panel:
used from Biolegend:
Brilliant Violet 650™ anti-human CD223 (LAG-3) Antibody (Clone: 11C3C65, Cat #369315) 1:200 dilution ,
PE/Fire™ 700 anti-human CD127 (IL-7Rα) (Clone: A019D5, Cat #351365), 1:100 dilution
PE/Fire™ 810 anti-human TIGIT (VSTM3) Antibody (Clone: A15153G, Cat #372745) 1:200 dilution
PE/Dazzle™ 594 anti-T-bet Antibody (Clone: 4B10, Cat #644827) 1:400 dilution
Alexa Fluor® 700 anti-human TNF-α Antibody (Clone: MAb11, Cat #502927) 1:400 dilution
used from BD Biosciences:
BV605 Mouse Anti-Human CD279 (PD-1) (Clone EH12.1, Catalog No: 563245), 1:200 dilution
Purified Mouse Anti-Human CD8 (Clone RPA-T8, Catalog No: 555364), 1:200 dilution
PE-Cy™5 Mouse Anti-Human CD161 (Clone DX12, Catalog No: 551138), 1:100 dilution
BUV563 Mouse Anti-Human CD4 (Clone SK3, Catalog No: 612912), 1:200 dilution
BUV737 Mouse Anti-Human TIM-3 (CD366) (Clone 7D3, Catalog No: 7488820), 1:100 dilution
BUV805 Mouse Anti-Human CD3 (Clone UCHT1, Catalog No: 612895), 1:200 dilution
BV421 Mouse Anti-Human CD152 (Clone BNI3, Catalog No: 562743), 1:100 dilution
BV480 Mouse Anti-Human CD45RA (Clone HI100, Catalog No: 566114), 1:200 dilution
BB700 Mouse Anti-Human CD69 (Clone FN50, Catalog No: 747520), 1:200 dilution
PE-Cy7 Mouse Anti-Human CD25 (Clone 2A3, Catalog No: 51-9016097) 1:200 dilution
Alexa Fluor® 647 Mouse Anti-Human CD197 (CCR7) (Clone 150503, Catalog No: 560816) 1:100 dilution
APC-H7 Mouse Anti-Human CD45RO (Clone UCHL1, Catalog No: 561137) 1:200 dilution
BUV395 Mouse Anti-Human IFN-γ (Clone B27, Catalog No: 563563) 1:400 dilution
BV750 Mouse Anti-Human Granzyme B (Clone GB11, Catalog No: 624380) 1:400 dilution
BV785 Mouse Anti-TCF-7/TCF-1 (Clone S33-966, Catalog No:624292) 1:200 dilution
used from ThermoFisher:
KLRG1 Monoclonal Antibody (13F12F2), Alexa Fluor™ 488 (Catalog # 53-9488-42) 1:100 dilution
IL-2 Monoclonal Antibody (MQ1-17H12), eFluor™ 450 (Catalog # 48-7029-42) 1:200 dilution
TOX Monoclonal Antibody (TXRX10), PE (Catalog # 12-6502-82) 1:200 dilution

For the mouse panel:
used from Biolegend:
Spark PLUS UV395™ anti-mouse CD25 Antibody (Clone: PC61, Cat #102083) 1:200 dilution
Brilliant Violet 570™ anti-mouse Ly-6C Antibody (Clone: HK1.4, Cat #128029) 1:200 dilution
Brilliant Violet 605™ anti-mouse/human CD44 Antibody (Clone IM7, Cat #103047) 1:200 dilution
PE/Dazzle™ 594 anti-mouse CD152 Antibody (Clone: UC10-4B9, Cat #106317) 1:200 dilution
Alexa Fluor® 700 anti-mouse CD62L Antibody (Clone: MEL-14, Cat #104426)1:200 dilution
FITC anti-mouse CD127 (IL-7Rα) Antibody (Clone: A7R34, Cat #135007) 1:100 dilution
PerCP anti-mouse CD45 Antibody (Clone: 30-F11, Cat #103129) 1:200 dilution
APC/Cyanine7 anti-mouse IFN-γ Antibody (Clone: XMG1.2, Cat #505849) 1:400 dilution
used from BD Biosciences:
BUV496 Hamster Anti-Mouse TCR β Chain (Clone H57-597,) 1:200 dilution
BV421 Mouse Anti-Mouse TIGIT (Clone 1G9, Catalog No: 565270) 1:200 dilution
BV480 Mouse Anti-Mouse CD366 (TIM-3) (Clone 5D12, Catalog No: 747618) 1:200 dilution
BV650 Rat Anti-Mouse CD223 (Clone C9B7W, Catalog No: 740560) 1:200 dilution
BV711 Rat Anti-Mouse CD279 (PD-1) (Clone 29F.1A12, Catalog No: 568563) 1:200 dilution
BV750 Rat Anti-Mouse TNF (Clone MP6-XT22, Catalog No: 566365) 1:400 dilution

Alexa Fluor® 647 Mouse Anti-Mouse RORγt (Clone Q31-378, Catalog No: 562682) 1:200 dilution
PE Mouse Anti-TCF-7/TCF-1 (Clone S33-966, Catalog No: 564217) 1:200 dilution
APC-R700 Rat Anti-Mouse IL-2 (Clone JES6-5H4, Catalog No: 565186) 1:200 dilution
used from ThermoFisher:
KLRG1 Monoclonal Antibody (2F1), PE-Cyanine5.5 (Catalog # 35-5893-82) 1:200 dilution
EOMES Monoclonal Antibody (Dan11mag), PE-Cyanine5 (Catalog # 15-4875-82) 1:200 dilution

T Cell Activation and Immunotherapy Antibodies
InVivoMAb anti-mouse PD-1 (CD279), BioXcell, Clone: RMP1-14, Catalog #BE0146
InVivoMAb rat IgG2b isotype control, anti-keyhole limpet hemocyanin, BioXcell, Clone: LTF-2, Catalog #BE0090
Ultra-LEAF™ Purified anti-mouse CD3ε Antibody, BioLegend, Clone: 145-2C11, Cat #100340
Ultra-LEAF™ Purified anti-mouse CD28 Antibody, BioLegend, Clone: 37.51, Cat #102116
Ultra-LEAF™ Purified anti-human CD3 Antibody, BioLegend, Clone: OKT3, Cat #317326
Ultra-LEAF™ Purified anti-human CD28 Antibody, BioLegend, Clone: CD28.2, Cat #302934

Confocal and Immunoblotting (IB) Antibodies
ND1 Polyclonal antibody, proteintech, Cat no : 19703-1-AP 1:500 dilution
ND2 Polyclonal antibody, proteintech, Cat no : 19704-1-AP 1:500 dilution
MT-ND5 Polyclonal antibody, proteintech, Cat no : 55410-1-AP 1:500 dilution
NDUFA5 Polyclonal antibody, proteintech, Cat no : 16640-1-AP 1:500 dilution
Anti-beta Actin antibody [AC-15] ab6276 1:20000 dilution
NFAT1 (D43B1) XP® Rabbit mAb #5861, Cell Signaling Technology 1:100 dilution

Validation

All commercially available antibodies used in this study were validated by the manufacturers and detailed in their respective product sheets. Validation data and additional antibody information can be accessed by searching the catalog numbers provided on the manufacturers' websites.
https://www.biolegend.com/ja-jp
https://www.bdbiosciences.com/en-us
https://www.thermofisher.com/tw/zt/home.html
https://bioxcell.com/
https://www.ptglab.com/
https://www.cellsignal.com/
https://www.abcam.com/en-us

Eukaryotic cell lines

Policy information about cell lines and Sex and Gender in Research

Cell line source(s)

B16-F10 (ATCC CRL-6475) are provided by Dr. Chia-Rui Shen (Ya-Shan Chen et al. Hum Gene Ther. 2019 Mar). MTCQ1 generated and provided by Dr. Shu-Chun Lin(Yi-Fen Chen et al. Oral Oncol. 2019 Aug)

Authentication

Cell lines used in this study were provided by other research groups. These cell lines were not authenticated in the current study.

Mycoplasma contamination

The cell lines used in this study were not tested for Mycoplasma contamination.

Commonly misidentified lines
(See ICLAC register)

No commonly misidentified cell lines, as defined by the ICLAC register, were used in this study.

Animals and other research organisms

Policy information about studies involving animals; ARRIVE guidelines recommended for reporting animal research, and Sex and Gender in Research

Laboratory animals

Tcrb KO (Strain #:002118) and OT-1(Strain #:003831) were from The Jackson Laboratory and keep in . C57BL/6JNarl was purchased from National Laboratory Animal Center. Mice were housed in National Laboratory Animal Center or conventional animal facilities at Laboratory Animal Center, Chang Gung Memorial Hospital, Linkou, Taiwan. In all animal experiments, mice aged 6–8 weeks were used, with sex-matching applied across each experiment, including both male and female subjects. The following strains were utilized. C57BL/6J mice, They were kept in individually ventilated cages under standard 12-hour light/dark cycles and maintained at an ambient temperature of 19–23 °C.

Wild animals

No wild animals were involved in this study.

Reporting on sex

Both male and female animals were included in the experiments, and data were analyzed to account for sex-based differences where relevant. Sex was considered during the experimental design to ensure balanced representation in all groups.

Field-collected samples

No samples were collected from the field.

Ethics oversight

All experiments involving laboratory animals followed the guidelines for animal experiments of Chang Gung Memorial Hospital (CGMH) and were approved by the IACUC of CGMH.

Note that full information on the approval of the study protocol must also be provided in the manuscript.

Clinical data

Policy information about clinical studies

All manuscripts should comply with the ICMJE guidelines for publication of clinical research and a completed CONSORT checklist must be included with all submissions.

Clinical trial registration	The study protocol was approved by the Medical Ethics and Human Clinical Trial Committee of Chang Gung Memorial Hospital (IRB No. 202400085B0) and is registered on ClinicalTrials.gov (ID: NCT06676293).
Study protocol	This single-center, prospective salvage therapy trial was conducted under compassionate-use conditions. The protocol details can be accessed upon request from the Medical Ethics and Human Clinical Trial Committee of Chang Gung Memorial Hospital.
Data collection	Patients with refractory nasopharyngeal carcinoma (NPC) who had not responded to standard treatment and anti-PD-1 therapy were recruited between August 2023 and March 2024 at the Oncology Department of Linkou Chang Gung Memorial Hospital. Nine patients were screened, and eight patients were randomized to receive either combination therapy (L-asparaginase and anti-PD1) or anti-PD1 monotherapy. Data collection included PET/CT imaging, biochemical lab tests, and EBV DNA titer monitoring. Additionally, blood was drawn from each patient, and peripheral blood mononuclear cells (PBMCs) were isolated for flow cytometry analysis, while serum was collected for metabolomic analysis.
Outcomes	Patients were monitored through routine clinical assessments, including PET/CT imaging, biochemical lab tests, and EBV DNA titer analysis. Primary and secondary outcome measures included the assessment of tumor response to therapy, measured by PET/CT and RECIST criteria for the primary outcome. Changes in biochemical lab parameters, EBV DNA levels, and patient survival rates were recorded as secondary outcomes. These outcomes were pre-defined and analyzed as described in the protocol.

Plants

Seed stocks	*Report on the source of all seed stocks or other plant material used. If applicable, state the seed stock centre and catalogue number. If plant specimens were collected from the field, describe the collection location, date and sampling procedures.*
Novel plant genotypes	*Describe the methods by which all novel plant genotypes were produced. This includes those generated by transgenic approaches, gene editing, chemical/radiation-based mutagenesis and hybridization. For transgenic lines, describe the transformation method, the number of independent lines analyzed and the generation upon which experiments were performed. For gene-edited lines, describe the editor used, the endogenous sequence targeted for editing, the targeting guide RNA sequence (if applicable) and how the editor was applied.*
Authentication	*Describe any authentication procedures for each seed stock used or novel genotype generated. Describe any experiments used to assess the effect of a mutation and, where applicable, how potential secondary effects (e.g. second site T-DNA insertions, mosiacism, off-target gene editing) were examined.*

Flow Cytometry

Plots

Confirm that:

☒ The axis labels state the marker and fluorochrome used (e.g. CD4-FITC).

☒ The axis scales are clearly visible. Include numbers along axes only for bottom left plot of group (a 'group' is an analysis of identical markers).

☒ All plots are contour plots with outliers or pseudocolor plots.

☒ A numerical value for number of cells or percentage (with statistics) is provided.

Methodology

Sample preparation	Cells were harvested from tumors using mechanical homogenization and digestion in collagenase type I (Gibco) and DNase (Roche) in RPMI for 30 min at 37 °C. Tumor mixtures and spleens were dissociated through a 70-µm filter and washed with PBS. Tumor suspensions were pelleted and resuspended in 40% Percoll solution, underlaid with 80% Percoll in a 15 mL conical flask. After centrifugation at 2000 g for 30 min, the middle layer was removed, washed in PBS, and counted.
Instrument	Flow cytometry was performed on a BD FACSymphony™ A5 Cell Analyzer or BD FACSVerse™ Cell Analyzer
Software	FlowJo software (version 10.8.1)
Cell population abundance	Naïve CD8+ T cells were isolated with naïve CD8 adverse selection cocktail and LS columns (Miltenyi) from C57BL/6 wild-type mice splenocytes and lymph nodes.
Gating strategy	FSC-SSC-H gating was used as the preliminary strategy to identify the lymphocyte population. Subsequent analysis focused on effector T cells (CD8+) across all experiments. Each cell population was further gated based on the expression of surface or intracellular markers, as detailed in the manuscript.

☒ Tick this box to confirm that a figure exemplifying the gating strategy is provided in the Supplementary Information.
