# Supplementary figures and images for: Asparagine deprivation enhances T cell antitumour response in patients via ROS-mediated metabolic and signal adaptations
Source: Nat Metab. 2025 Mar 5;7(5):918–27. doi: 10.1038/s42255-025-01245-6 (PMC12116382; doi:10.1038/s42255-025-01245-6)

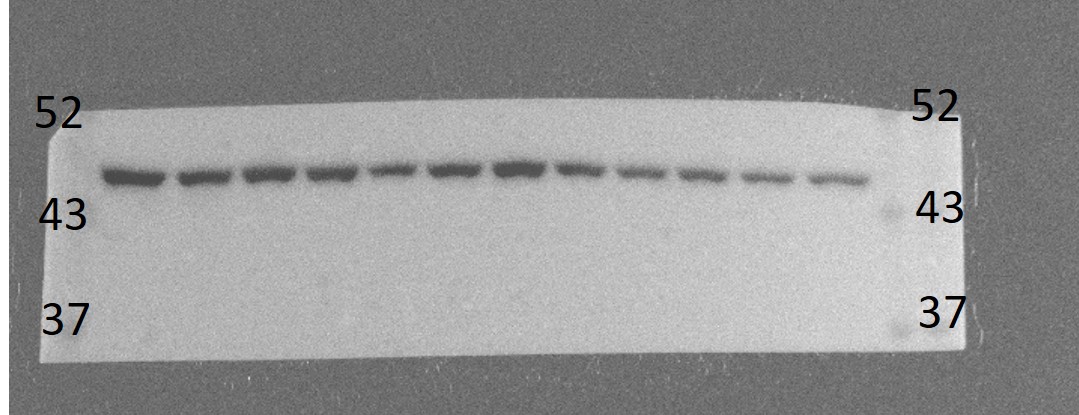

Supplement: Supplementary file 4 — Western blot raw data. [file 42255_2025_1245_MOESM4_ESM.jpg]

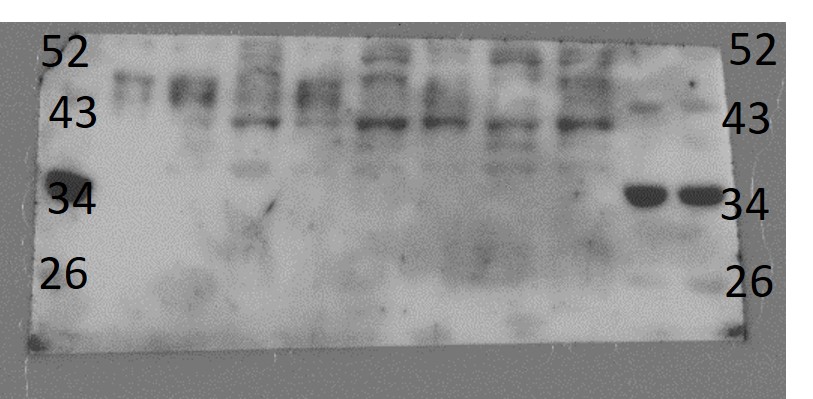

Supplement: Supplementary file 5 — Western blot raw data. [file 42255_2025_1245_MOESM5_ESM.jpg]

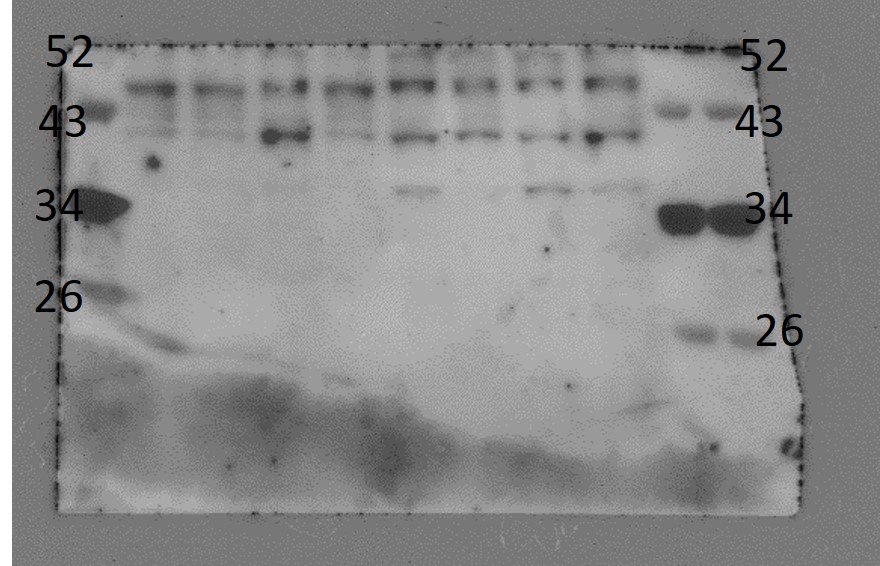

Supplement: Supplementary file 6 — Western blot raw data. [file 42255_2025_1245_MOESM6_ESM.jpg]

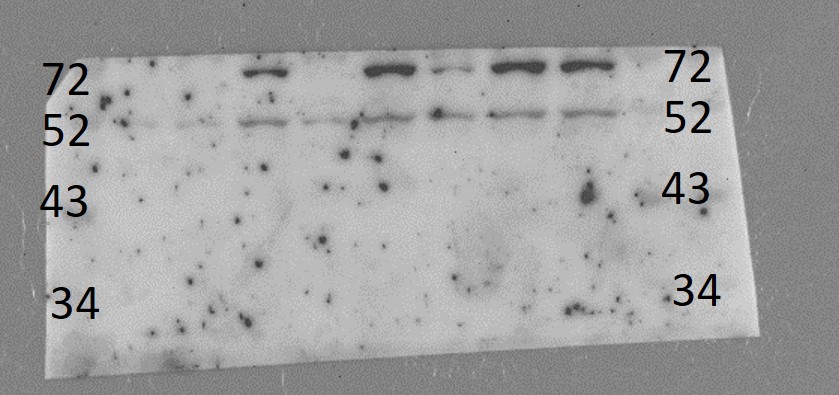

Supplement: Supplementary file 7 — Western blot raw data. [file 42255_2025_1245_MOESM7_ESM.jpg]
