# Supplementary material for: Author Correction: Asparagine deprivation enhances T cell antitumour response in patients via ROS-mediated metabolic and signal adaptations
Source: Nat Metab. 2026 Sep 15;8(10):2202. doi: 10.1038/s42255-026-01633-6 (PMC13690589; doi:10.1038/s42255-026-01633-6)
Supplement: Supplementary file 1 [file 42255_2026_1633_MOESM1_ESM.pdf]

nature metabolism

Supplementary information

Author Correction: Asparagine deprivation enhances T cell antitumour response in patients via ROS-mediated metabolic and signal adaptations

In the format provided by the authors and unedited

Original, uncorrected figure

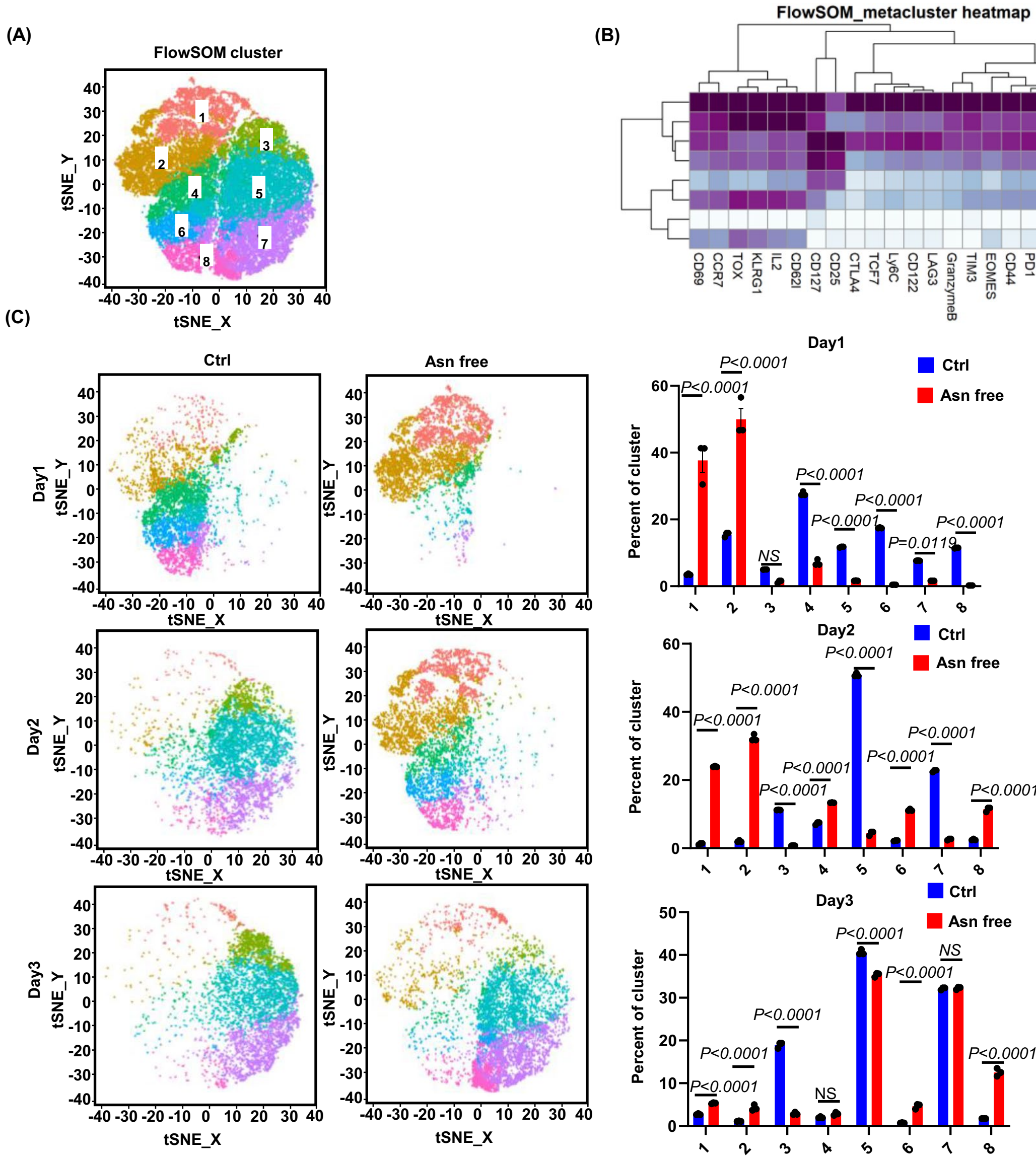

Extended Data Fig. 5
